# Assessing Observable Resolution in Open-Access sEMG Datasets for High-Resolution Hand-Gesture Research: A Quantization-Aware Methodological Study

**DOI:** 10.3390/s26216890

**Published:** 2026-10-30

**Authors:** Alexandra Gomez-Caraveo, Miguel E. Bravo-Zanoguera, José A. Amezquita-García, Guillermo Galaviz, Fabian N. Murrieta-Rico

**Affiliations:** 1 Facultad de Ingeniería, Universidad Autónoma de Baja California, Mexicali 21100, Baja California, Mexico; alexandra.gomez@uabc.edu.mx (A.G.-C.); alejandro.amezquita@uabc.edu.mx (J.A.A.-G.); ggalaviz@uabc.edu.mx (G.G.); 2 Ingeniería Mecatrónica, Universidad Politécnica de Baja California, Mexicali 21376, Baja California, Mexico; fnmurrietar@upbc.edu.mx

**Keywords:** surface electromyography (sEMG), public datasets, quantization, effective resolution, data integrity, data traceability

## Abstract

**Highlights:**

**What are the main findings?**

- Recordings analyzed from five of the eleven public sEMG datasets exhibited a preserved observable quantization grid, whereas recordings from six exhibited disrupted numerical structures.
- Grid alignment (Egrid) quantified alignment with the inferred numerical grid, while zero differences and signal cardinality provided complementary indicators.

**What are the implications of the main findings?**

- Declared ADC resolution and numerical precision of distributed data do not necessarily reflect the quantization structure retained in public datasets.
- Quantization auditing improves data traceability for studies comparing signal resolution, acquisition systems, and post-acquisition processing.

**Abstract:**

Open-access surface electromyography (sEMG) datasets are widely used for gesture-recognition research; however, limited documentation of acquisition and post-processing may prevent verification of whether the numerical structure imposed during acquisition is preserved in the distributed data. This study evaluates one selected signal vector from each of eleven public forearm sEMG datasets using a quantization-aware framework, complemented by an assessment across participants and channels to examine within-dataset consistency. The framework distinguishes declared resolution (DR) from observable effective resolution (OER), a signal-dependent descriptor of the numerical granularity observable in the distributed data rather than a measure of hardware ENOB. The analysis uses the minimum nonzero first-order difference (Δ_min_), zero-difference proportion (P_0_), relative signal cardinality, and a quantization-grid alignment metric (Egrid) to characterize numerical discreteness and grid preservation. Recordings analyzed from five datasets exhibited ordered difference levels consistent with a preserved observable quantization grid, whereas those from six showed disrupted structures. The complementary assessment revealed both consistent numerical patterns and within-dataset variability across the examined recordings. Egrid quantified approximate grid alignment at the adopted tolerance, while P0 and cardinality served as complementary descriptors. Validation using an independently documented 24-bit dataset yielded Egrid = 1, despite low P0 and high cardinality, demonstrating that these indirect descriptors depend on signal and acquisition characteristics. The results show that nominal analog-to-digital converter (ADC) resolution and stored numerical precision alone cannot establish acquisition-level quantization integrity. Numerical auditing can therefore provide additional traceability when public datasets are used to investigate signal resolution and quantization effects.

## 1. Introduction

In recent years, leading semiconductor manufacturers have promoted commercial solutions specifically designed for biomedical measurements, including 24-bit ADCs and analog front-end devices for biopotential signal acquisition (e.g., EEG, ECG, and EMG) [1,2]. However, technological constraints in wearable devices for handling high-resolution biosignals raise the question of whether such resolution can be effectively exploited in algorithms for sEMG signal analysis, interpretation, and application. Given their accessibility, cost-effectiveness, and widespread reuse, open-access datasets represent a practical resource for investigating this question without requiring new acquisition of comparable multisubject recordings. However, their numerical representation does not necessarily retain traceability to the original acquisition process or preserve the quantization characteristics implied by the reported ADC resolution. Therefore, before assessing the potential benefits of high-resolution sEMG data in subsequent applications, it is necessary to determine whether the distributed signals retain an observable discrete structure consistent with the documented acquisition characteristics. In this study, traceability refers operationally to the extent to which documented acquisition characteristics can be related to numerical properties that remain observable in the distributed signal; it does not imply metrological traceability established through a calibration chain.

In this context, the term “high-resolution sEMG” requires careful interpretation. High bit-depth acquisition and high-precision numerical representation are not equivalent: a signal originally acquired with a high-resolution ADC may subsequently be converted to floating-point format and subjected to digital processing, potentially altering or obscuring its original quantization structure. Consequently, the numerical precision of a publicly distributed dataset cannot, by itself, establish traceability to the nominal resolution of the acquisition system. Biosignal datasets do not reflect the performance of the analog-to-digital converter (ADC) in isolation; rather, they describe the cumulative response of the entire acquisition chain: sensor + analog front-end + ADC/sampling + digital processing + storage. In this pipeline, the physiological signal undergoes successive transformations before being deposited into the database, see Figure 1.

Contemporary sEMG acquisition systems continue to employ moderate ADC resolutions, particularly in embedded and wearable implementations. Modern wearable ExG front-ends increasingly integrate medium-to-high-resolution digitization with power-efficient circuit architectures [3] A recent review of sEMG acquisition systems reported 10 bits as the most frequently used resolution among the examined designs, followed by 12 and 24 bits, with implementations relying on either microcontroller-integrated or external ADCs [4]. The practical benefit of increasing bit depth, however, remains application-dependent. Classical considerations based primarily on signal-to-noise ratio have suggested limited benefits from increasing resolution beyond moderate bit depths [5]. More recently, Verdini et al. [6] experimentally compared 8- and 12-bit sEMG acquisition for recognition of 20 hand gestures and found that ADC resolution had only a marginal effect on classification performance under the evaluated conditions, whereas sampling frequency had a more consistent influence. Similarly, Ortiz-Catalán et al. [7] reported diminishing returns beyond approximately 14 bits for conventional myoelectric features. Shinde et al. [8], using synthetic and real-world EMG data, reported diminishing improvements in reconstruction error beyond approximately 8–10 bits and identified this range as a favorable compromise between signal fidelity and data efficiency; they also observed that classification performance reached a plateau at a lower bit depth for the specific task evaluated. Taken together, these findings do not establish a universal resolution requirement for sEMG, but rather indicate that the usefulness of additional bit depth depends on the acquisition conditions, signal characteristics, processing chain, and intended application. This diversity of acquisition and processing architectures further motivates numerical auditing of distributed datasets when ADC resolution or quantization is itself the variable under investigation.

Although not all biosignal applications require higher ADC resolutions, 24-bit converters are increasingly used in modern acquisition systems to provide high dynamic range and signal fidelity. Modern clinical EEG amplifiers commonly employ 24-bit digitization [9], while wireless EEG systems span resolutions from 12 to 24 bits [10]. High-bit-depth acquisition has also been incorporated into recent wearable and multichannel EMG systems [11,12], illustrating the growing technical feasibility of 24-bit biosignal acquisition. However, this technological availability is not equally reflected in open-access sEMG datasets, where high-resolution recordings remain scarce and the reported ADC resolution does not necessarily guarantee that the original quantization structure remains observable in the distributed data. This limitation restricts the traceability and suitability of such datasets for studies aimed at evaluating the effects of acquisition resolution. A preliminary study by the authors introduced this problem through an exploratory analysis of quantization integrity in two public sEMG datasets [13]. The present study substantially extends that preliminary work by evaluating eleven datasets, formalizing the distinction between declared and observable effective resolution, incorporating complementary numerical indicators of grid alignment and signal characteristics, and providing methodological validation using controlled and independently documented reference signals.

Accordingly, this study addresses two main research questions: to what extent does documented acquisition resolution remain reflected in the observable numerical structure of open-access sEMG datasets, and does an observable discrete quantization structure remain identifiable or show evidence of alteration potentially associated with post-acquisition processing? Focusing on forearm recordings relevant to hand-gesture applications and their associated documentation, a quantization-aware methodological framework is introduced to compare reported acquisition specifications with the numerical structure of the distributed signals and estimate their observable effective resolution directly from the data. The methodology is further evaluated using a controlled numerical reference with a known discrete structure and an independently documented PhysioNet dataset acquired with a 24-bit ADC. Rather than benchmarking classification performance as a function of ADC resolution, this study provides reproducible criteria for assessing quantization integrity and identifying suitable datasets for future controlled investigations of the potential benefits of high-resolution sEMG acquisition.

## 2. Materials and Methods

### 2.1. Dataset Characterization and Descriptive Parameters

Publicly available sEMG datasets exhibit limited standardization in the reporting of acquisition parameters, particularly regarding quantization characteristics and traceability to the original analog-to-digital conversion stage. Existing datasets originate from heterogeneous acquisition systems with varying hardware configurations, including differences in channel count, sampling frequency, front-end filtering, and nominal ADC resolution. This heterogeneity introduces challenges for reproducibility, comparability, and the interpretation of signal resolution and quality.

The public sEMG datasets included in this study were selected according to the following criteria: (i) recordings acquired from forearm muscles during hand gesture tasks, (ii) availability of acquisition system information (e.g., recording device or nominal ADC resolution), and (iii) sufficient documentation to partially reconstruct the original acquisition conditions. Sufficient documentation was defined as the availability of information allowing the relevant acquisition characteristics, including the recording system and nominal ADC resolution, to be identified or independently verified from the associated publication, repository documentation, or manufacturer specifications. Although these criteria were applied, several datasets do not provide complete traceability to the original ADC configuration, limiting direct assessment of the quantization characteristics imposed during acquisition.

A total of eleven publicly available datasets were selected. Table 1 summarizes the datasets, corresponding authors, data sources, and associated references or access links. For convenience, each dataset was assigned a unique dataset identifier (DB1–DB11), which is used consistently throughout this paper to simplify references and comparisons among datasets.

The dataset identifiers are independent of the original repository names and were introduced solely to provide a concise notation for the analyses, figures, and statistical comparisons presented in the following sections.

Table 2 summarizes the primary applications for which the selected public sEMG datasets were originally developed, as reported in their corresponding publications or repository descriptions. The original application of the datasets was not required to be exclusively hand-gesture classification, provided that the recordings involved forearm sEMG acquired during hand-gesture or related motor tasks and satisfied the acquisition-documentation criteria described above.

Despite the diversity of their original applications, the present study focuses exclusively on assessing the preservation of the discrete quantization structure of the distributed sEMG signals. One selected sEMG channel from each dataset was used for the quantization analysis. Because the present analysis concerns the observable numerical structure of the distributed signals rather than application-specific classification performance, differences in the original application scope did not preclude their inclusion.

Table 3 summarizes the reported acquisition conditions and gesture protocols of the selected datasets, highlighting the heterogeneity of the instrumentation and the varying level of documentation available across datasets. In particular, differences in ADC resolution, sampling frequency, signal conditioning, and preprocessing limit direct comparison of the reported acquisition characteristics and motivate the subsequent assessment of the numerical structure of the distributed sEMG signals.

The parameters considered in the analysis were grouped into three categories: (i) dataset descriptors, (ii) acquisition-system specifications, and (iii) metrics derived from the distributed signals. Accordingly, Table 4 summarizes the reported and empirically derived parameters considered relevant for characterizing each dataset and assessing its quantization properties. Dataset descriptors include information such as repository location and file format, whereas acquisition specifications comprise the documented sampling rate, acquisition device, and declared resolution (DR), when available. The observable effective resolution (OER) and other quantization-related metrics were derived directly from the numerical structure of the distributed data and are formally defined in the subsequent subsections. Baseline noise RMS and range-to-noise ratio (RNR) are additionally included as complementary signal descriptors to support interpretation of the acquisition conditions and numerical characteristics of each dataset.

### 2.2. Resolution Definitions and Observable Metrics

Given the heterogeneity and limited traceability identified in Section 2.1, direct hardware-based assessment of resolution is generally not feasible for publicly available datasets. Accordingly, this study distinguishes three complementary notions of resolution. The declared resolution (DR) refers to the nominal resolution reported in the dataset documentation, associated publication, or acquisition-device specifications, and represents the nominal quantization capability attributed to the acquisition system. The effective number of bits (ENOB), in contrast, is conventionally determined from controlled hardware measurements of ADC dynamic performance and cannot be reliably reconstructed from publicly released datasets lacking the required calibration and acquisition-chain information. Furthermore, the term “high-resolution sEMG” may refer either to high-bit-depth acquisition or to high-precision numerical representation, although these represent fundamentally different sources of signal granularity. To address this distinction, this study introduces the observable effective resolution (OER), defined as the empirically measurable numerical discreteness of the distributed signal and estimated from the minimum nonzero absolute difference between consecutive samples. Because this spacing may be affected by numerical transformations, finite-precision representation, or post-acquisition processing, OER should not be interpreted as a direct measurement of the original ADC quantization step. The minimum nonzero difference (Δ_min_) represents the smallest observable numerical spacing in the distributed signal, whereas the observable effective resolution (OER) expresses this spacing relative to the available signal range in equivalent bits. OER is therefore a descriptor of the observable numerical granularity of the distributed signal and should not be interpreted as ADC resolution, ENOB, or numerical storage precision.

Conventional amplitude-distribution analysis alone is insufficient to determine whether the original discrete quantization structure has been preserved. In particular, histogram binning may aggregate multiple quantization levels and thereby obscure the underlying numerical lattice, allowing signals with different quantization structures to exhibit similar amplitude distributions. Therefore, the proposed framework combines complementary numerical descriptors with direct analysis of the quantization grid. The evaluated parameters include signal cardinality, first-difference distributions, the proportion of exact zero differences (P_0_), minimum nonzero spacing (Δ_min_), and quantization-grid alignment (Egrid). Observed signal range, baseline noise RMS, and range-to-noise ratio (RNR) are additionally considered as contextual signal descriptors. The definitions and calculation procedures for these metrics are presented in the subsequent subsections.

### 2.3. Resolution Estimation from Distributed Data

The observable numerical structure of each dataset was analyzed from the distributed signal (x[n]), regardless of its storage data type. The underlying principle is that a preserved observable discrete grid produces first differences aligned with integer multiples of a fundamental spacing, whereas the occurrence of intermediate off-grid differences indicates disruption of that numerical structure. A first-difference sequence was computed as

(1) $$\Delta x\left[n\right]=x\left[n+1\right]-x\left[n\right].$$

The minimum nonzero absolute difference,

(2) $$\Delta_{\min}=\min\{|\Delta x[n]|:|\Delta x[n]|\neq 0\}$$

was defined as the minimum observable numerical spacing in the distributed signal. Zero differences were identified using exact numerical equality in the distributed representation, without applying an additional tolerance. This choice preserves small nonzero differences as part of the observable numerical structure rather than reassigning them to an assumed original quantization level.

The observed signal range was calculated as

*Robs* = *x*_max_ − *x*_min_, (3)

and the observable effective resolution (OER), expressed in equivalent bits, was estimated as

(4) $$OER=log_{2}\left(\frac{Robs}{\Delta_{\min}}\right)$$

As discussed in Section 2.2, OER characterizes the numerical discreteness observable in the released data and should not be interpreted as a direct measurement of the original ADC resolution. OER was calculated using the observed signal range for all datasets. Because $R_{\mathrm{obs}}$ is signal-dependent and may occupy only a fraction of the ADC full-scale input range, OER values below DR do not necessarily indicate loss of acquisition resolution. For DB1, which provides a documented full-scale input range, an additional comparison was performed using the measured Δ_min_ to assess its consistency with the declared ADC resolution.

To further characterize the observable discrete structure of the distributed signals, the first-difference distribution, proportion of exact zero differences ($P_{0}$), and signal cardinality were evaluated. Relative signal cardinality was defined as the number of unique numerical values observed in the analyzed signal vector divided by the total number of samples (N), expressed as a percentage. Repeated difference values and integer multiples of $\Delta_{\min}$ provide evidence of a preserved observable numerical grid, whereas irregular intermediate spacing may reflect numerical transformations occurring after A/D conversion. Throughout this study, the terms preserved and disrupted grid refer to the observable numerical structure of the analyzed distributed recordings and do not imply direct verification of the original ADC quantization lattice.

Quantization-grid alignment was quantitatively evaluated using the grid-alignment metric $E_{grid}$. For each nonzero difference,

(5) $$r\left[n\right]=\frac{\left|\Delta x\left[n\right]\right|}{\Delta_{\min}}$$

and alignment with the inferred uniform lattice was determined from

(6) $$\left|r\left[n\right]-\mathrm{round}\left(r\left[n\right]\right)\right|\leq \epsilon$$

where (ϵ = 0.001), empirically selected in the present analysis as an operational tolerance for assessing proximity to the nearest integer multiple of $\Delta_{\min}$. The grid-alignment metric was then calculated as the proportion of nonzero differences satisfying this condition:

(7) $$E_{\mathrm{grid}}=\frac{N_{\mathrm{aligned}}}{N_{\Delta \neq 0}},$$

where $N_{\mathrm{aligned}}$ is the number of nonzero differences satisfying Equation (6), and $N_{\Delta \neq 0}$ is the total number of nonzero differences. All nonzero differences in the analyzed vector were included; no separate exclusion criterion was applied to extreme values or potential artifacts. Values of Egrid close to unity indicate strong alignment with the inferred quantization lattice, whereas lower values indicate increasing occurrence of off-grid numerical values. The selected tolerance is used as a descriptive analysis parameter and is not proposed as a theoretically derived or universally optimal value.

The analysis was conducted at two complementary levels. First, one continuous recording vector was selected from each dataset for detailed characterization of its observable numerical structure, including first-difference distributions and examination of the ten smallest nonzero difference levels, which are reported in Appendix A. Because the objective was to characterize numerical discretization rather than inter-subject, inter-channel, or classification variability, the selected recording was used as a numerical probe of the documented acquisition and data-distribution chain. For a recording containing *N* samples, the first-difference analysis evaluates *N* − 1 consecutive sample transitions. These transitions are not treated as statistically independent observations but provide repeated opportunities to observe the numerical structure present in the analyzed recording.

The subject and recording for the detailed analysis were selected primarily on the basis of practical accessibility within each repository, using the first readily accessible recording suitable for analysis, without screening or optimization based on the resulting numerical metrics. When multiple sEMG channels were available, the first accessible channel was generally used. For DB1, channel 4 was selected because the other available channels exhibited a marked DC offset. The exact file and signal analyzed for each dataset are reported in Table 5. All numerical analyses were performed using MATLAB R2026a (The MathWorks, Inc., Natick, MA, USA).

Second, to assess within-dataset consistency, the same numerical indicators were calculated for additional recordings from up to five participants and three channels per participant, where available. Exceptions were DB4, for which eight channels from a single participant were examined, and DB8, for which two channels per participant were available. This complementary assessment was intended to examine the consistency of the observed numerical characteristics across selected recordings, rather than to establish their uniformity throughout the entire datasets. The additional results are presented in Appendix B.

### 2.4. Noise and Signal-Range Considerations

Noise conditions and signal-range utilization were considered as complementary descriptors of the acquisition characteristics. Baseline noise was estimated as the RMS amplitude of a visually selected portion within resting events identified as such in the available dataset documentation or recording protocol. Because the original datasets did not follow a common baseline-acquisition protocol, this measurement should be interpreted as a contextual descriptor of the combined contributions of acquisition electronics, the electrode–skin interface, physiological fluctuations, and environmental interference present in the distributed signal, rather than as an independent measurement of acquisition-system noise.

Rather than conventional SNR, which requires an appropriate definition of signal power, a range-to-noise ratio (RNR) was used as a descriptive metric:

(8) $$RNR_{dB}=20log_{10}\left(\frac{R_{obs}}{RMS_{noise}}\right)$$

where $R_{obs}=x_{max}-x_{min}$ represents the amplitude range occupied by the analyzed signal.

Baseline noise and RNR were not used as direct criteria for quantization-grid preservation. Analog noise may increase the number of occupied ADC codes and reduce repeated consecutive values without necessarily disrupting the underlying discrete lattice. Therefore, these descriptors were used to contextualize the observed P_0_, cardinality, and resolution estimates, whereas Egrid was used to directly assess alignment with the inferred quantization grid. RNR is used only as a descriptive range-to-baseline-noise measure and should not be interpreted as conventional SNR. Because its numerator is based on the observed maximum-to-minimum range, it may be sensitive to extreme values, while the baseline RMS reflects the noise and other fluctuations present in the selected resting interval.

### 2.5. Methodological Validation Using Controlled and Independent Reference Signals

As an elementary controlled verification of the proposed numerical metrics, an 8-bit triangular sequence was generated directly in unsigned integer (uint8) representation. The sequence spanned the complete code range from 0 to 255 and back to 0 and was repeated for three periods. Consecutive samples therefore differed by a known unit spacing (q=1). The signal was subsequently converted to double-precision floating-point representation, and the same $\Delta_{\min}$, OER, and $E_{\mathrm{grid}}$ calculations described above were applied. This test was designed to verify the behavior of the proposed metrics under a known discrete numerical structure, rather than to reproduce the physiological characteristics of sEMG. A complementary controlled experiment was conducted to illustrate how digital filtering may alter or preserve the numerical structure of a quantized signal. A synthetic signal was quantized to 8 bits and subsequently processed using a floating-point band-pass FIR filter and an integer-coefficient FIR operation. Changes in Δ_min_, P0, relative cardinality, and Egrid, together with deviations from the original quantization grid, were evaluated. Experimental details and MATLAB code are provided in the Supplementary Materials.

As an independent real-biosignal reference, a documented 24-bit physiological dataset was also analyzed to evaluate the proposed methodology under high-resolution acquisition conditions. The Evoked Auditory Responses in Normals dataset available on PhysioNet was used as an independent reference [36,37]. The dataset contains simultaneously recorded auditory brainstem response (ABR) and otoacoustic emission (OAE) signals acquired at 48 kHz using a LynxTWO sound card. The LynxTWO sound card employed AKM AK5394 24-bit A/D converters with a reported dynamic range of 123 dB [38]. For ABR acquisition, the electrode signal was band-pass filtered from 30 to 3000 Hz and amplified by a factor of 50,000 using a GRASS QP511 Quad AC Amplifier (Grass Technologies, West Warwick, RI, USA) before digitization. Although these signals differ physiologically from sEMG, the dataset provides documented 24-bit acquisition and was therefore used as a reference for evaluating whether the proposed metrics can identify a preserved discrete numerical structure under independently documented high-resolution acquisition conditions.

The distributed recordings contain synchronized ABR and OAE signals in WFDB format and were analyzed in physical units using the same numerical procedures described in Section 2.3 and Section 2.4. The validation examined the minimum nonzero spacing Δ_min_, OER, first-difference structure, P_0_, signal cardinality, and Egrid. Emphasis was placed on whether nonzero differences remained aligned with integer multiples of Δ_min_, as quantified by Egrid. The independent ABR/OAE dataset was not intended as a physiological model of sEMG or as validation of sEMG-specific ADC quantization behavior. Rather, it served as a real-biosignal reference for assessing whether the proposed framework can identify an observable discrete numerical structure in recordings obtained through an independently documented 24-bit acquisition chain.

## 3. Results

### 3.1. Analyzed Signals and Observable Resolution

For each public dataset, one continuous sEMG recording vector was selected for numerical analysis, following the procedure described in Section 2.3. Table 5 identifies the specific file and signal channel analyzed for each dataset, providing traceability between the reported results and the distributed data. The complete available recording vector was used in each case, without discarding any samples, to characterize its observable numerical structure.

Figure 2 presents the selected sEMG signals analyzed from the eleven public datasets listed in Table 5. The recordings exhibit different temporal characteristics according to their respective acquisition protocols, ranging from repeated gesture sequences to individual gesture executions or complete movement cycles. Despite these differences, the present analysis focuses exclusively on the numerical structure of the distributed signals and their observable quantization characteristics.

Following the methodology described in Section 2.3, the minimum nonzero spacing (Δ_min_) was obtained from the first-difference sequence, and the observable effective resolution (OER) was estimated relative to the observed signal range. Table 6 summarizes the sampling frequency, acquisition device, declared resolution (DR), and estimated OER for the selected recordings from the eleven datasets. The results show that OER coincides with DR for some analyzed recordings, whereas substantial discrepancies are observed for others.

These discrepancies should not be interpreted as changes in the physical resolution of the acquisition hardware. OER values below DR are not necessarily indicative of degraded acquisition, since the analyzed signal may occupy only a fraction of the available ADC input range. An OER approaching DR indicates extensive utilization of the available numerical range, although saturation can only be established from additional evidence of clipping or repeated limiting values. Conversely, OER values exceeding DR indicate numerical spacing finer than expected from the declared acquisition resolution and therefore cannot be attributed to the nominal ADC quantization alone, potentially reflecting post-acquisition numerical processing. Accordingly, the DR–OER comparison provides an initial assessment of the numerical resolution observable in the distributed signals, whereas preservation of the underlying quantization grid is evaluated separately in Section 3.2.

### 3.2. Quantization-Grid Preservation

To examine preservation of the quantization grid beyond the OER estimates, the first-difference structure of each selected recording was analyzed. For each signal, the frequency of exact zero differences (Δx = 0) was determined, followed by examination of the first nine distinct nonzero difference levels, including $\Delta_{\min}$. The smallest nonzero value corresponds to $\Delta_{\min}$, whereas subsequent levels were examined to determine whether they occurred as integer multiples of (Δ_min_), consistent with a preserved observable quantization grid. The complete difference levels and their occurrence frequencies for each dataset are provided in Appendix A.

Table 7 summarizes the observed difference-level patterns. The first nine nonzero difference levels (including $\Delta_{\min}$) were reported as a compact inspection of the local numerical structure near the minimum observable spacing. This subset was not used as the sole basis for grid-preservation assessment; $E_{\mathrm{grid}}$ evaluates alignment over all nonzero differences in the analyzed vector. The recordings analyzed from DB1, DB3, DB4, DB7, and DB9 exhibited an ordered structure dominated by integer multiples of $\Delta_{\min}$, whereas the recordings analyzed from DB2, DB5, DB6, DB8, DB10, and DB11 showed irregular or intermediate difference levels inconsistent with a preserved uniform grid.

Figure 3 and Figure 4 illustrate these two behaviors using the first ten difference levels. Figure 3 shows the analyzed recordings exhibiting an ordered quantization-grid structure, whereas Figure 4 shows recordings with disrupted spacing patterns. In contrast to conventional amplitude histograms, which primarily describe the amplitude distribution of the sEMG signals, the first-difference histograms expose the numerical spacing between stored values and therefore provide a more direct visualization of the underlying discrete structure.

Quantization-grid preservation was subsequently characterized using the metrics defined in Section 2.3. Table 8 reports Δ_min_, the proportion of exact zero differences (P_0_), the quantization-grid alignment metric (Egrid), and relative signal cardinality for each analyzed recording. These descriptors exhibited different sensitivities to the observable numerical structure and were therefore interpreted jointly with the difference-level patterns rather than as independent classification criteria. In particular, $E_{\mathrm{grid}}$ quantifies approximate alignment with the inferred lattice at the adopted tolerance, whereas $P_{0}$ and relative cardinality characterize sample repetition and numerical occupancy, respectively.

The complementary within-dataset assessment revealed both consistency and variability in the observable numerical characteristics of the evaluated recordings (Appendix B). DB1, DB3, DB4, and DB7 exhibited consistent minimum nonzero spacing (Δ_min_) and Egrid = 1 across the examined participants and channels. In contrast, several datasets showed low grid-alignment values or variations in numerical descriptors across recordings. Notably, DB9 exhibited participant-dependent differences, with some recordings retaining an aligned discrete structure and others showing disrupted numerical patterns. These findings indicate that the numerical characteristics observed in an individual recording may recur across multiple recordings but are not necessarily uniform throughout a dataset.

### 3.3. Signal-Range and Noise Characteristics

Table 9 summarizes the signal-range and noise characteristics obtained for the eleven analyzed datasets, including the maximum and minimum signal amplitudes, observed signal range (Robs), baseline noise RMS, and range-to-noise ratio (RNR). Baseline noise and RNR could not be estimated for datasets in which suitable resting intervals were unavailable or insufficient for baseline-noise estimation.

The RNR values varied considerably across the analyzed datasets, reflecting differences in signal-range utilization and baseline noise conditions. These parameters were considered complementary descriptors of the distributed signals and were not used as direct indicators of quantization-grid preservation. Their relationship with the observed quantization characteristics is considered in the Discussion.

### 3.4. Validation with Controlled and Independent Reference Signals

The controlled 8-bit numerical signal contained 1531 samples and 1530 consecutive differences, all of which were either +1 or −1. The analysis therefore recovered $\Delta_{\min}=1$, equal to the known unit spacing imposed in the sequence. With $R_{\mathrm{obs}}=255$, the resulting OER was 7.9944 bits, consistent with the known 8-bit numerical representation. All nonzero differences were exact integer multiples of $\Delta_{\min}$, yielding $E_{\mathrm{grid}}=1.000$ for ε=0.001. These results provide a controlled verification of the behavior of the proposed metrics when the underlying discrete numerical structure is known a priori.

In the complementary filtering experiment, the floating-point band-pass FIR filter increased relative signal cardinality from 2.55% to 100% and reduced Egrid from 1 to 0.0018, indicating substantial disruption of the original observable numerical structure. In contrast, the integer-coefficient FIR operation preserved the original minimum spacing and yielded Egrid = 1. The corresponding maximum deviations from the original quantization grid were approximately 0.5 and 0 LSB, respectively. Detailed results are provided in the Supplementary Materials.

The reference Evoked Auditory Responses in Normals dataset was analyzed using the same procedures applied to the public sEMG datasets. Figure 5 shows enlarged views of the first-difference distributions for the selected ABR and OAE recording. A minimum nonzero spacing of Δ_min_ = 1.19 × 10^−8^, was obtained for the OAE channel, and the first nonzero difference levels occurred as successive integer multiples of Δ_min_. The corresponding Egrid = 1 indicates complete alignment of the analyzed differences with the inferred uniform quantization grid.

Table 10 summarizes the OER estimated for the ABR and OAE channels of three participants. The estimated values ranged from 15 to 23 bits for ABR and from 22 to 23 bits for OAE. Although the acquisition system was documented as providing 24-bit recordings, the observable resolution varied between signals and participants, illustrating that OER characterizes the numerical resolution observable in the distributed signal rather than the nominal resolution of the acquisition hardware.

Notably, the same $\Delta_{\min}$ was observed across the three OAE recordings, whereas two of the three ABR recordings shared the same minimum spacing, providing an additional indication of consistency in the observable numerical structure across participants.

The complementary indicators for the selected OAE recording are reported in Table 11. Despite the preserved grid alignment Egrid = 1, the signal exhibited a low zero-difference proportion (P_0_ = 0.0251%) and a relative cardinality of 18.5%. Thus, these results demonstrate that a preserved quantization grid can coexist with a low proportion of repeated consecutive samples and relatively high signal cardinality.

## 4. Discussion

The results reveal that the nominal resolution of the acquisition system, the numerical resolution observable in the distributed data, and preservation of the original quantization structure represent related but distinct properties. Agreement between declared resolution (DR) and observable effective resolution (OER) was observed for several datasets, whereas others exhibited substantial discrepancies. Importantly, an OER equal to or greater than the DR does not by itself demonstrate preservation of the original ADC quantization structure. OER characterizes the numerical spacing observable in the released signal, whereas quantization-grid preservation requires determining whether such spacing remains organized according to a consistent discrete lattice. This distinction is particularly relevant for public datasets distributed in floating-point formats, where scaling, filtering, normalization, or other numerical transformations may introduce values between the original acquisition levels.

The first-difference analysis provided a direct means of examining this distinction. Analyzed recordings exhibiting a preserved observable grid showed difference levels predominantly aligned with integer multiples of $\Delta_{\min}$, whereas recordings exhibiting disrupted structures contained intermediate or irregularly spaced values. Among the evaluated indicators, Egrid directly quantified approximate alignment with the inferred lattice at the adopted tolerance, whereas the zero-difference proportion P_0_ and relative signal cardinality characterize repetition and occupancy of numerical values. These descriptors were therefore interpreted jointly with the observed difference-level structure rather than as independent classification criteria. This distinction is illustrated by DB10, for which RMS-based signal conditioning produces a high proportion of repeated values and relatively low cardinality despite the absence of consistent alignment with the inferred quantization grid. Thus, repeated values alone should not be interpreted as evidence that acquisition-level quantization has been preserved.

Taken together, the evaluated indicators provided complementary information about the observable numerical structure of the analyzed recordings, but no individual descriptor was sufficient to distinguish all preserved and disrupted grid patterns. $P_{0}$ and relative signal cardinality characterize sample repetition and numerical occupancy, respectively, but both depend on signal and acquisition characteristics. $E_{\mathrm{grid}}$ quantifies approximate alignment with integer multiples of $\Delta_{\min}$ at the adopted tolerance; however, DB2 yielded $E_{\mathrm{grid}}=1$ despite exhibiting an irregular difference-level pattern. Conversely, DB10 showed substantial sample repetition and relatively low cardinality despite a disrupted grid. The descriptors were therefore interpreted jointly with the observed difference-level structure, as summarized in Table 12.

The independent 24-bit reference dataset provides important context for this limitation. Although the ABR and OAE recordings produced OER values ranging from 15 to 23 bits and 22 to 23 bits, respectively, the analyzed OAE signal retained a regular integer lattice with Egrid = 1. At the same time, it exhibited a very low P_0_ (0.0251%) and relatively high cardinality (18.5%). These results demonstrate that low sample repetition and high code occupancy can coexist with complete alignment to an observable quantization grid. Consequently, P_0_ and cardinality depend not only on quantization preservation but also on biosignal dynamics and the complete acquisition chain. In the reference system, the 24-bit AK5394 converter, its approximately 123-dB specified dynamic range, 50,000-fold front-end gain, 30–3000 Hz bandwidth, and 48-kHz sampling rate provide conditions under which small physiological and instrumental variations can occupy many adjacent numerical levels without disrupting the underlying lattice. These observations further support interpreting the numerical descriptors jointly and in the context of the signal and acquisition conditions, rather than using any individual descriptor as a standalone criterion for quantization-grid preservation.

The individual datasets also illustrate why acquisition resolution and distributed numerical representation should not be treated as interchangeable. DB2 provides a particularly clear example: the acquisition-device documentation indicates a 12-bit ADC, whereas the distributed recordings use a signed 16-bit representation and yield an OER of approximately 16 bits. Because the available documentation does not specify how the 12-bit ADC output was mapped to the 16-bit WFDB representation, the latter should not be interpreted as evidence of 16-bit physical A/D conversion. DB11 presents a different traceability problem. Although its acquisition system reports 16-bit EMG signal resolution, the distributed data exhibit much finer numerical spacing and do not preserve the corresponding discrete grid. The documented Trigno architecture includes sensor-side conditioning, wireless transmission, a base station, and host-computer processing, but the available information does not identify the stage at which the observed numerical transformation occurred. The DB11 MATLAB files additionally store the sEMG values in single-precision floating-point format; however, the storage type alone does not establish whether or at what stage the observed grid disruption was introduced. Similarly, for DB8, although the ADC is documented, incomplete information on the acquisition and processing chain prevents retrospective identification of the origin of the observed numerical structure. These cases emphasize that knowledge of the nominal ADC or acquisition-device resolution alone is insufficient to reconstruct the numerical history of a publicly distributed signal.

Importantly, documented filtering did not necessarily result in loss of the observable quantization grid. Among the signals analyzed in this study, DB4 was reported as band-pass filtered before storage and analysis, yet it still exhibited an ordered discrete structure. The available documentation was insufficient to determine how this structure was retained through the processing chain. The effect of filtering on the observable numerical grid therefore depends on its implementation and cannot be inferred from the reported use of filtering alone [39]. The complementary controlled experiment further illustrates this distinction: floating-point band-pass filtering disrupted the observable numerical grid of the synthetic signal, whereas an integer-coefficient FIR operation preserved it. These results demonstrate possible processing-dependent changes in numerical granularity without establishing which processing stages, if any, caused the alterations observed in the public datasets. Post-acquisition processing does not necessarily imply that a dataset is unsuitable for its intended application. Filtering, normalization, rectification, RMS processing, or floating-point representation may be appropriate—and potentially beneficial—for feature extraction or classifier development. However, such processing changes the interpretation of numerical values when the objective is to compare acquisition resolutions or investigate quantization effects. A processed dataset may therefore remain highly useful for gesture recognition while no longer retaining sufficient information to reconstruct its acquisition-level quantization structure. Conversely, preservation of a quantization grid should not be interpreted as evidence of superior classification performance or higher physiological information content. These are separate questions. Determining whether controlled changes in bit depth or numerical densification affect downstream classification performance therefore requires separate experiments in which these factors are systematically varied while the underlying signals and classification conditions are held constant.

The signal-range and noise results further illustrate why nominal ADC bit depth alone does not determine the usable information contained in a recording. The measured RNR values indicate that, in several datasets, the separation between the occupied signal range and baseline noise is substantially smaller than the dynamic range theoretically associated with high-resolution conversion. Under such noise-limited conditions, increasing ADC bit depth does not necessarily provide a proportional increase in usable physiological resolution, since the finer quantization steps may become small relative to the noise imposed by the complete acquisition chain. Likewise, increasing the numerical precision of the stored data through floating-point representation cannot by itself add physiological information. RNR and quantization-grid metrics should therefore be regarded as complementary descriptors: the former characterizes signal-range utilization relative to baseline noise, whereas the latter evaluates the numerical structure retained in the distributed data.

These observations have practical implications for the use of open-access sEMG datasets. Public datasets are commonly reused for algorithm development, classifier comparison, and evaluation of sensing strategies, often under the assumption that the distributed signals faithfully reflect the documented acquisition characteristics. When the processing history between the ADC and the released file is incompletely documented, however, nominal bit depth alone cannot establish that assumption. Preservation of the discrete structure is particularly important when the research question explicitly concerns ADC resolution, quantization, or comparisons between low- and high-resolution acquisition systems. In these applications, numerical auditing provides an additional level of traceability by distinguishing documented hardware specifications from properties that remain observable in the released data.

The complementary within-dataset assessment (Appendix B) further contextualizes the record-level findings. Several datasets exhibited consistent numerical characteristics across the examined participants and channels, whereas others showed variability, particularly DB9. This heterogeneity indicates that shared acquisition hardware or documented processing conditions do not necessarily ensure identical observable numerical structures among recordings from the same dataset. Accordingly, the detailed characterization of a selected recording should not be interpreted as establishing quantization-grid preservation or disruption throughout an entire dataset.

More generally, the results highlight the importance of documenting the complete data path from acquisition hardware to public distribution. Information on ADC resolution, input range, analog conditioning, digital filtering, scaling, normalization, data-type conversion, and file-generation procedures would substantially improve the reproducibility and interpretability of public biosignal datasets. The proposed framework does not reconstruct undocumented processing stages or establish the hardware ENOB from stored signals; rather, it provides a means of determining what numerical discretization remains observable after those stages. Although the detailed first-difference characterization was performed on one selected recording per sEMG dataset, the complementary assessment extended the numerical analysis to multiple participants and channels where available. This additional evaluation provides evidence of within-dataset consistency or variability among the examined recordings but does not establish statistical representativeness or uniformity across entire repositories. Recording-session variability was not systematically assessed. Nevertheless, in the independently documented reference dataset, the three evaluated OAE recordings exhibited the same Δ_min_, while two of the three ABR recordings shared the same value. Future work should further investigate within-dataset variability across additional recordings and sessions, extend the framework to other biosignal modalities and acquisition architectures, and investigate separately how controlled changes in bit depth and post-acquisition processing affect quantization-grid preservation and downstream machine-learning performance.

## 5. Conclusions

This study demonstrates that the declared resolution of an acquisition system and the numerical precision of a distributed sEMG dataset are not sufficient to establish the quantization structure observable in the distributed data. The proposed framework distinguishes observable effective resolution (OER) from quantization-grid preservation and combines first-difference analysis with complementary numerical descriptors, with Egrid providing a quantitative assessment of alignment with the inferred quantization grid. Analysis of selected recordings from eleven public sEMG datasets revealed both preserved and disrupted observable numerical structures, while validation using an independently documented 24-bit dataset provided complementary evidence that grid preservation can be identified even under signal and acquisition conditions producing low sample repetition and high cardinality. The complementary assessment across participants and channels further revealed both consistency and variability in observable numerical characteristics, highlighting the limitations of extrapolating record-level findings to entire datasets. These findings emphasize the importance of documenting the complete data path from acquisition to public distribution. Numerical auditing of open-access datasets can therefore provide an additional level of traceability for studies involving signal resolution, quantization, and comparison of acquisition systems.

## Figures and Tables

**Figure 1:**
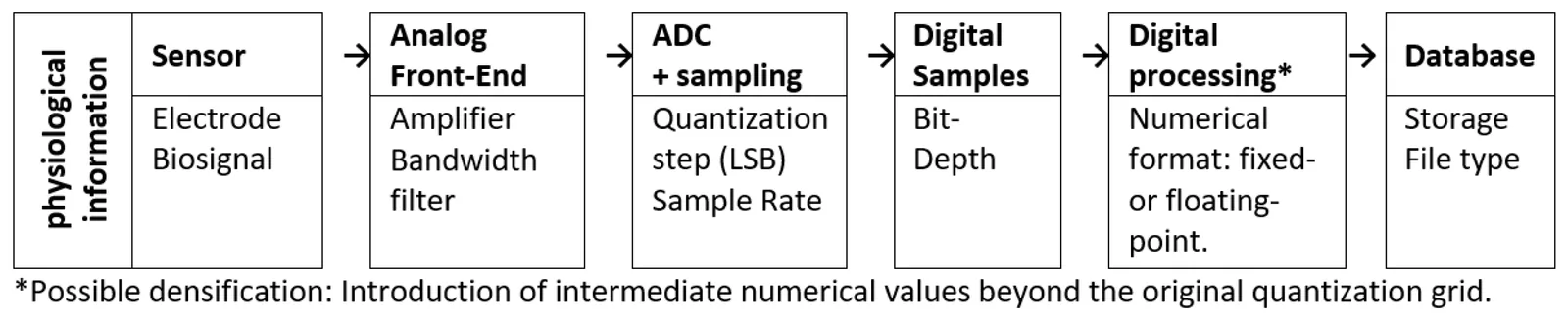
Acquisition and processing pipeline of physiological signals. The resulting dataset reflects the integrated contribution of the full chain: sensor (biosignal electrode), analog front-end, ADC and sampling, digital processing, and final database storage.

**Figure 2:**
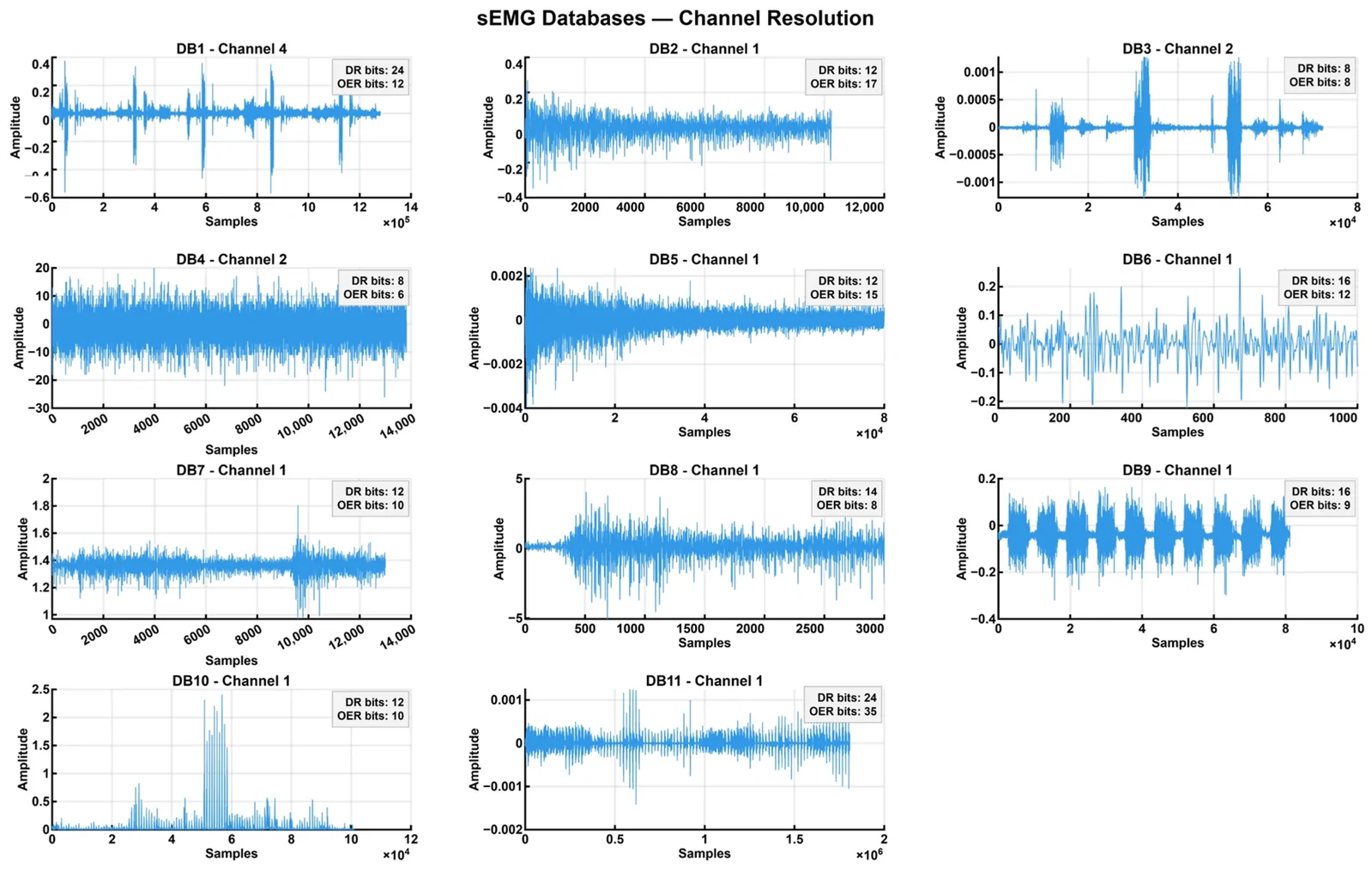
Selected sEMG signals analyzed from the eleven public datasets. The recordings illustrate the diversity of signal patterns and acquisition protocols, including repeated gesture sequences, individual movements, and nonperiodic activity. Horizontal scales correspond to the complete selected recordings and therefore differ among datasets.

**Figure 3:**
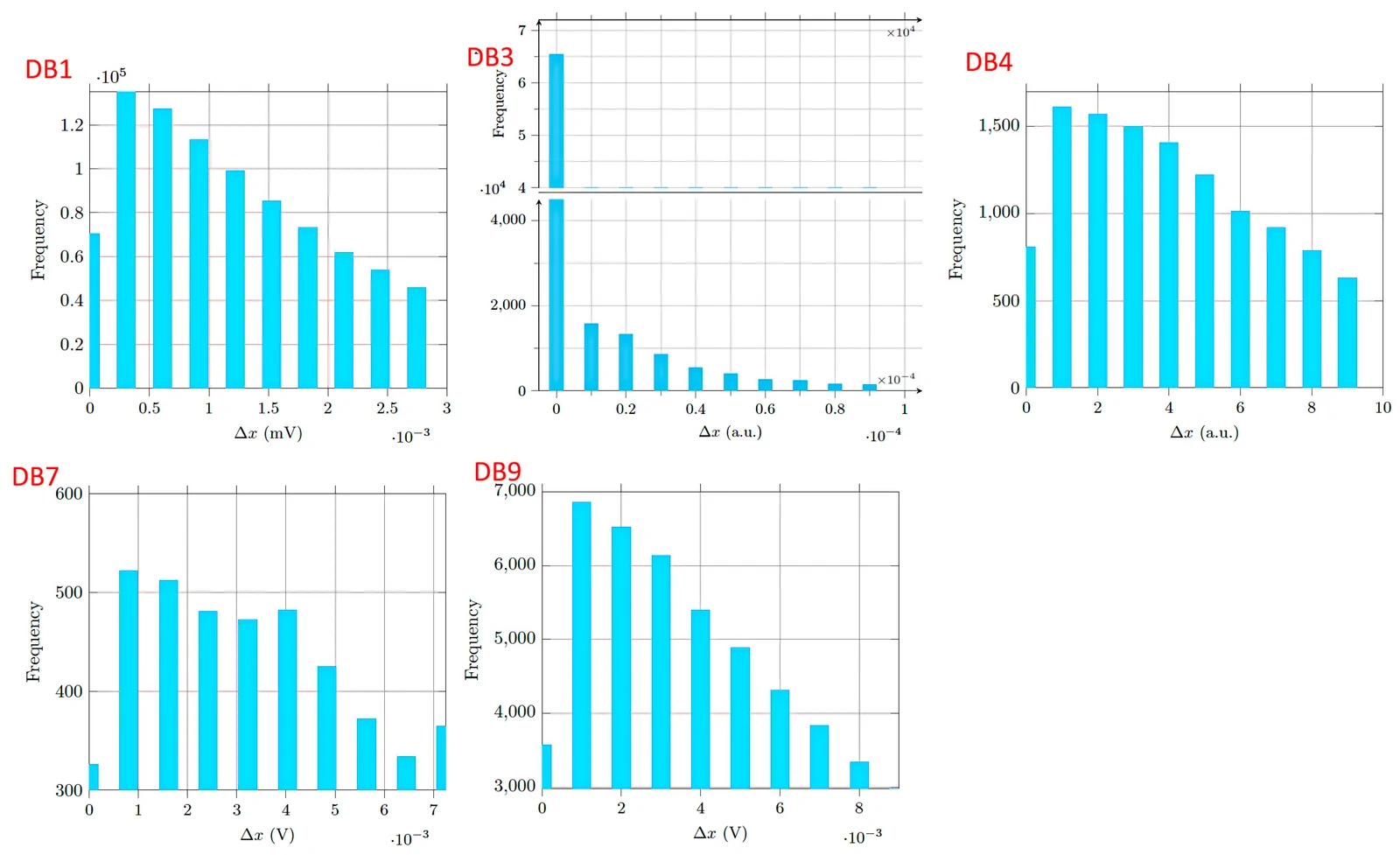
Histograms of the first ten first-order difference levels for recordings from datasets exhibiting an ordered quantization-grid structure (DB1, DB3, DB4, DB7, and DB9). The difference levels occur predominantly as integer multiples of Δ_min_, consistent with preservation of the observable quantization grid.

**Figure 4:**
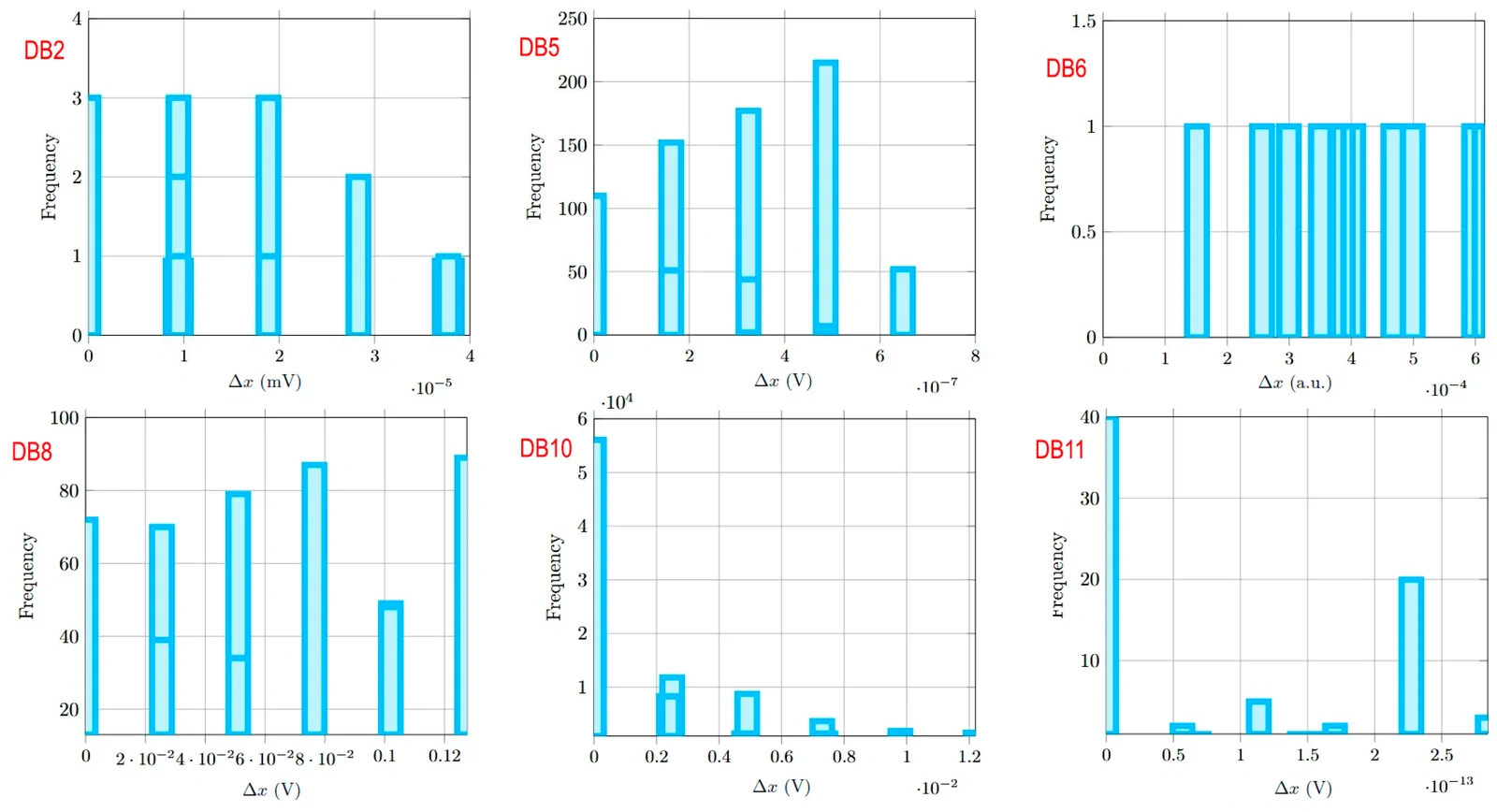
Histograms of the first ten first-order difference levels for recordings from datasets exhibiting a disrupted quantization-grid structure (DB2, DB5, DB6, DB8, DB10, and DB11). Intermediate values and irregular spacing prevent the formation of a consistent sequence of integer multiples of Δ_min_.

**Figure 5:**
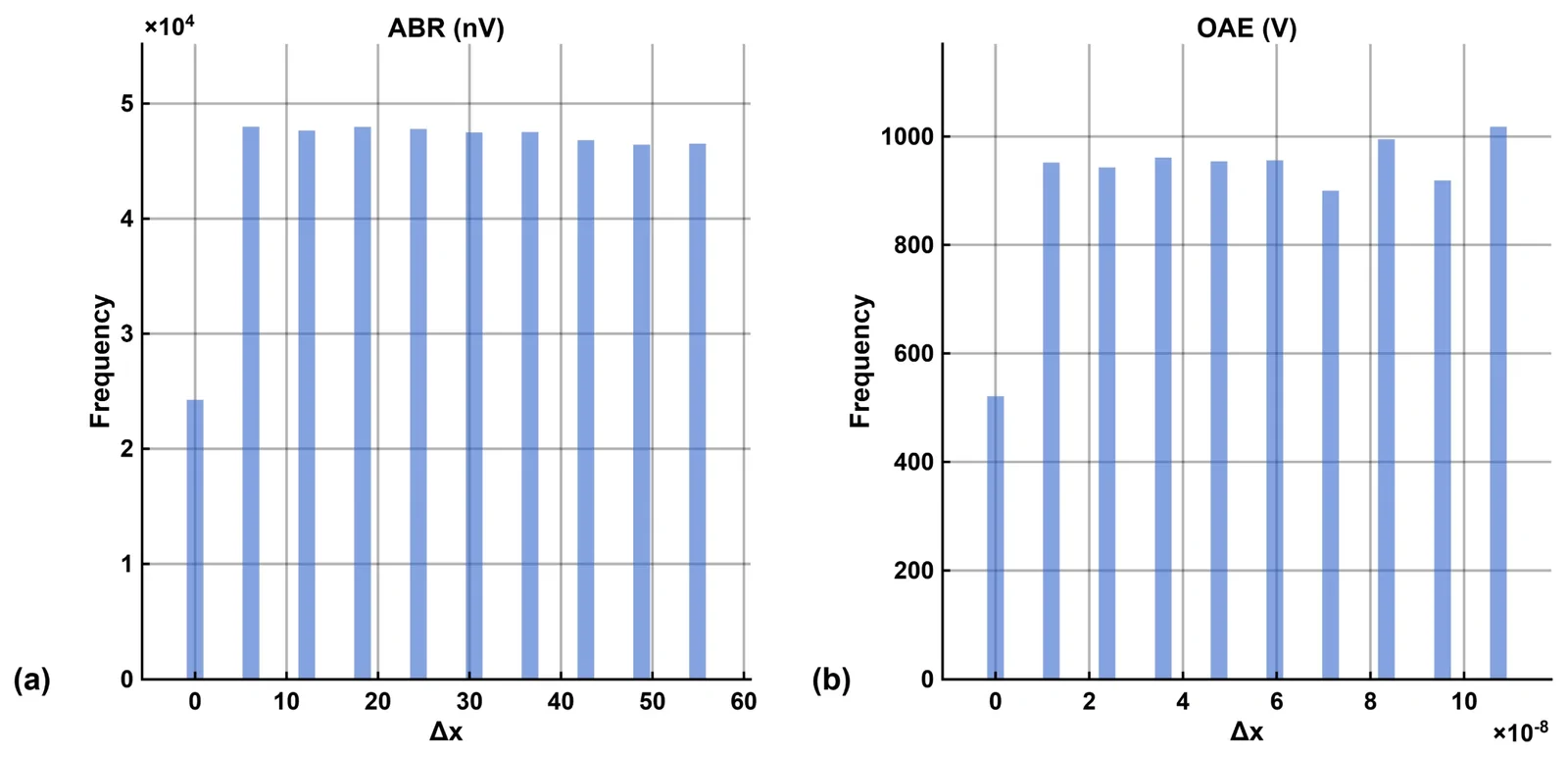
First-difference distribution of the selected ABR and OAE recording from the 24-bit reference dataset. (**a**) ABR channel; (**b**) OAE channel. The enlarged view shows successive integer multiples of Δ_min_, consistent with preservation of the inferred quantization grid.

**Table 1:** Public sEMG datasets selected for analysis, with their assigned dataset identifiers, authors, and corresponding data sources.

Dataset ID	Dataset/Author(s)	Source
DB1	Ozdemir et al.	Mendeley Data [14]; associated publication [15]
DB2	Pradhan et al.	PhysioNet [16]; associated publication [17]
DB3	Krilova et al.	UCI Machine Learning Repository [18]
DB4	Khan et al.	Mendeley Data [19]; associated publication [20]
DB5	Khushaba et al.	Biosignals Repository [21]
DB6	Geng et al.	CapgMyo-DBa figshare Dataset [22]; associated publication [23]
DB7	Gomez-Correa et al.	springernature.figshare collections [24]; associated publication [25]
DB8	Sapsanis et al.	UCI Machine Learning Repository [26]
DB9	Miljković et al.	zenodo.org [27]
DB10	Atzori et al.	Ninapro public database [28]; associated publication [29]
DB11	Atzori et al.	Ninapro public database [30]

**Table 2:** Original intended applications of the public sEMG datasets included in this study.

Dataset ID	Original Intended Application
DB1	Hand gesture recognition and EMG-based human–computer interaction.
DB2	Hand gesture recognition, biometrics, and electrode-shift robustness.
DB3	Analysis of factors affecting sEMG-based human–machine interface performance.
DB4	Assistive human–computer interfaces for users with physical or cognitive disabilities.
DB5	Myoelectric prosthetic finger control and EMG feature reduction.
DB6	High-density sEMG gesture recognition for real-time muscle–computer interfaces.
DB7	Hand gesture recognition, rehabilitation assessment, and prosthetic/orthotic control.
DB8	Daily hand grasp recognition for prosthetic hand control.
DB9	Electrode displacement effects on hand movement classification.
DB10	Benchmarking myoelectric movement classification for hand prostheses.
DB11	Development of naturally controlled non-invasive robotic hand prostheses.

**Table 3:** Reported acquisition conditions and gesture protocols of the selected public sEMG datasets.

No.	Sensor-Level Acquisition and Gesture Capture Protocol Information
DB1	Four-channel forearm sEMG acquired using a BIOPAC MP36 system (BIOPAC Systems Inc., Goleta, CA, USA) with Ag/AgCl bipolar surface electrodes 3M Red Dot (3M, Saint Paul, MN, USA), at a sampling frequency of 2 kHz and an amplitude range of ±10 mV. The acquisition system incorporates a 24-bit Cirrus Logic CS5351 ADC (Cirrus Logic Inc., Austin, TX, USA). The dataset provides raw and online-filtered signals (6th-order Butterworth bandpass, 5–500 Hz; 50 Hz notch); raw signal analyzed in this study. The protocol comprised 10 hand gestures, with five repetitions from 40 participants. Data are provided as MATLAB files (version R2026a, The MathWorks Inc., Natick, MA, USA) and CSV formats. ADC configuration and effective resolution at the dataset level were not explicitly reported.
DB2	EMG signals acquired using an EMGUSB2+ amplifier (OT Bioelettronica, Turin, Italy) with a gain of 500 and a sampling frequency of 2048 Hz. Twenty-eight monopolar pre-gelled Ag/AgCl electrodes were arranged in four rings around the forearm and wrist. Data were collected from 43 healthy participants over three sessions. The protocol comprised 16 hand and wrist gestures plus rest, with seven repetitions per gesture and 5 s recordings for each contraction. The device manual specifies a 12-bit A/D converter [31], whereas the PhysioNet data description reports signed 16-bit quantized values for the distributed Waveform Database Software Package (WFDB) files [17].
DB3	Eight-channel forearm sEMG acquired using a Myo armband (Thalmic Labs, Kitchener, ON, Canada) at a sampling frequency of 200 Hz and an 8-bit signal resolution. Raw sEMG data were collected from 36 participants performing two series of seven basic hand gestures. Each gesture was maintained for 3 s, followed by a 3 s rest period.
DB4	Eight-channel sEMG acquired from the upper limb of one participant using a Myo armband at a sampling frequency of 200 Hz. The signals were stored as signed 8-bit values and transmitted wirelessly via Bluetooth to a host computer. The protocol comprised four activities: rest, typing, push-ups, and lifting a heavy object. A 5–100 Hz band-pass filter was applied to the acquired sEMG signals before storage and analysis.
DB5	Eight-channel forearm sEMG acquired using a Delsys Bagnoli-8 system with DE 2.x series EMG sensors (Delsys Inc., Natick, MA, USA). The signals were amplified with a gain of 1000 and digitized at 4000 Hz with a reported 12-bit National Instruments data acquisition system. The acquired signals were band-pass filtered between 20 and 450 Hz, with a 50 Hz notch filter to suppress power-line interference. The protocol comprised 15 classes of individual and combined finger movements.
DB6	High-density sEMG acquired using a 128-channel (8 × 16) differential electrode array with silver wet electrodes. Signals were band-pass filtered between 20 and 380 Hz and sampled at 1000 Hz with 16-bit ADC conversion. The resulting values were normalized to the [−1, 1] range, corresponding to an input voltage range of [−2.5, 2.5] mV. The analyzed CapgMyo DB-a subset comprises eight isometric and isotonic hand gestures recorded from 18 participants.
DB7	Four-channel forearm sEMG acquired using the WyoFlex armband, a custom wearable device developed by the dataset authors (Instituto Politécnico Nacional, Mexico City, Mexico), at a sampling frequency of 1000 Hz. The acquisition system incorporates a 12-bit ADC implemented in a FireBeetle ESP32 microcontroller. Data were collected from 28 participants performing 10 wrist and grasping movements, with three repetitions per gesture. The four sEMG channels were distributed equidistantly around the forearm.
DB8	Two-channel sEMG acquired using differential EMG sensors connected to a Delsys Bagnoli Handheld EMG System (Delsys Inc., Natick, MA, USA). Signals were digitized using a NI USB-6009 data acquisition device, (National Instruments, Austin, TX, USA) which provides 14-bit analog-input resolution in differential mode [32,33], and recorded at a sampling frequency of 500 Hz using LabVIEW (National Instruments, Austin, TX, USA; version not reported by the dataset authors). The dataset documentation reports Butterworth band-pass filtering between 15 and 500 Hz and a 50 Hz notch filter, although the stage at which these filters were applied is not specified. The available documentation does not specify the NI USB-6009 input range used during acquisition. The protocol comprised six daily-life hand grasps performed by healthy participants.
DB9	Eight-channel forearm sEMG acquired using circular Ag/AgCl electrode pairs positioned circumferentially around the dominant forearm. Signals were amplified with a gain of 1000 and digitized using a National Instruments NI USB-6212 data acquisition device with 16-bit resolution at a sampling frequency of 1000 Hz. Data were collected from 10 able-bodied participants performing nine hand movements at three electrode-array positions.
DB10	Ten-channel sEMG acquired using Otto Bock MyoBock 13E200-50 electrodes, which provide an amplified, band-pass-filtered, and Root-Mean-Square (RMS) rectified output of the raw sEMG signal, with an amplification gain of approximately 14,000. The sensor outputs were acquired at 100 Hz using a National Instruments NI-DAQ PCMCIA 6024E data acquisition card with 12-bit resolution. The protocol comprised 53 movements, including rest, with 10 repetitions per movement. Therefore, the distributed signals correspond to sensor-conditioned rather than raw sEMG.
DB11	Twelve-channel sEMG acquired using Delsys Trigno Wireless sensors at a sampling frequency of 2 kHz and 16-bit EMG signal resolution [34,35]. The sensors provide an input range of approximately 11 mV, a gain of 909 ± 5%, and a bandwidth of approximately 20–450 Hz. Signals were transmitted wirelessly to the base station and transferred to the host computer via USB. The protocol comprised 50 movements, including rest, with six repetitions per movement. Data were distributed in MATLAB format, with sEMG values stored in single-precision floating-point representation.

**Table 4:** Dataset descriptors, acquisition-system specifications, and derived metrics considered for quantization assessment.

Parameter	Technical Description
Dataset ID	Dataset identifier (DB1–DB11) used throughout this study.
Reference/Author	Primary reference or author associated with the dataset.
Number of movements	Number of hand gestures or movements included in the dataset.
Channels	Number of simultaneously acquired sEMG channels.
Sampling frequency (Fs)	Sampling frequency used for signal acquisition, expressed in Hz.
Declared resolution (DR)	ADC resolution reported in the dataset documentation, associated publication, or acquisition-device specifications.
Samples analyzed (*N*)	Total number of samples in the selected signal vector used for the numerical analysis.
Minimum nonzero difference (Δ_min_)	Minimum nonzero absolute difference between consecutive samples in the distributed signal, obtained from (Δx[n] = ∣x[n + 1] − x[n]∣).
Maximum value (x_max_)	Maximum numerical value observed in the analyzed signal.
Minimum value (x_min_)	Minimum numerical value observed in the analyzed signal.
Observed dynamic range (R_obs_)	Numerical amplitude range of the analyzed signal, calculated as (x_max_ − x_min_).
Observable effective resolution (OER)	Observable numerical resolution estimated from $\Delta_{\min}$ and the observed signal range ($R_{\mathrm{obs}}$), expressed in bits. OER = log_2_(R_obs_/∆min)
Subjects	Number of subjects included in the dataset.
Acquisition device	Instrument, acquisition system, or device used to record the sEMG signals.
Baseline noise RMS	RMS value of the signal during the selected baseline or no-muscle-activity interval.
Observed signal range-to-noise ratio (RNR)	Ratio between the observed signal amplitude range and baseline noise RMS, expressed in dB. RNR_dB_ = 20log_10_((x_max_ − x_min_)/RMS_noise_)
Quantization-related metrics	Numerical metrics used to assess the observable discrete structure of the distributed signal, including P0, relative cardinality, and Egrid.
File format	Format in which the dataset is distributed (e.g., .mat, .dat, .txt, .xlsx, or .csv).

**Table 5:** File access path and sEMG signal selected for numerical analysis in each dataset.

ID	Analyzed File/Archive Path	Variable/Signal Analyzed
DB1	...\sEMG-dataset\raw\mat\1_raw.mat	data(:,4) ^1^
DB2	.../session1_participant1/session1_participant1_gesture1_trial1.dat	*rdsamp* (WFDB) ^2^ ≥ data(:,1)
DB3	emg+data+for+gestures.zip\EMG_data_for_gestures-master\02\1_raw_data_14-19_22.03.16.txt	*readmatrix* → data(:,2) ^3^
DB4	Dataset.zip\Dataset\subject_01\file_05\emg-1591011691.csv	*readmatrix* → data(:,2) ^4^
DB5	S1-Delsys-15Class.7z\S1-Delsys-15Class\HC_1.csv	*readmatrix* → data(:,1)
DB6	7210397\dba-s1.zip\ 001-001-001.mat\data	data(1,:)
DB7	\WyoFlex_Dataset\DIGITAL DATA\P1C1S1M1F1O1.txt	*readmatrix* → data(:,1)
DB8	semg+for+basic+hand+movements\Database 1\female_1.mat\cyl_ch1	cyl_ch1(:,1)
DB9	EMG dataset.7z\EMG dataset\ID1\ID1_3F_P1.txt	*readmatrix* → data(:,1)
DB10	\s1.zip\S1_A1_E1.mat\emg	emg(:,1)
DB11	\s1.zip\S1_E1_A1.mat\emg	emg(:,1)

^1^ Only sEMG channel 4 was analyzed. ^2^ Signal imported using *rdsamp* from the WFDB Software Package for MATLAB (version R2026a, The MathWorks Inc., Natick, MA, USA). ^3^ Channel 1 of the armband was analyzed. ^4^ EMG channel 1 of the armband was analyzed during rest.

**Table 6:** Acquisition characteristics, declared resolution (DR), and observable effective resolution (OER) for the selected recordings from the public sEMG datasets.

ID	*N*	Fs (Hz)	Acquisition Device	DR (Bits)	OER (Bits)
DB1	1,280,000	2000	BIOPAC MP36	24	12 *
DB2	10,240	2048	EMGUSB2	12 (ADC) 16 (WFDB representation)	16
DB3	72,322	200	MYO Thalmic	8	8
DB4	13,826	200	MYO Thalmic	8	6
DB5	80,000	4000	Delsys Bagnoli-8	12	15
DB6	1000	1000	Custom device	16	12
DB7	13,000	1000	WyoFlex armband	12	10
DB8	3000	500	NI USB-009	14	8
DB9	81,234	1000	NI6212	16	9
DB10	101,014	100	MyoBock 13E200-50 Ottobock/NI-daq PCMCIA 6024E	12	10
DB11	1,808,331	2000	Trigno Wireless electrodes	16	35

*: For DB1, OER was calculated from the observed signal range, as for all other datasets. Because the full-scale input range was also documented for DB1, an additional comparison was possible: when this range is used with the measured $\Delta_{\min}$, the resulting span corresponds to approximately 24 bits, consistent with the declared ADC resolution.

**Table 7:** Observed quantization-grid patterns based on the first nine nonzero difference levels, including Δ_min_, for the selected recording from each dataset. Complete difference values and occurrence frequencies are provided in Appendix A.

Dataset	Δx = 0	Δ_min_	Grid Pattern ^1^	Quantization Grid
DB1	70,416	3.0517578125 × 10^−4^	Integer multiples	Yes, Preserved
DB2	3	9.43599809671153 × 10^−6^	mostly irregular	Disrupted
DB3	65,381	1 × 10^−5^	Integer multiples	Yes, Preserved
DB4	807	1 × 10^0^	Integer multiples	Yes, Preserved
DB5	110	1.62 × 10^−7^	mostly irregular	Disrupted
DB6	0	1.51288651745074 × 10^−4^	irregular	Disrupted
DB7	326	8.05861 × 10^−4^	Integer multiples	Yes, Preserved
DB8	72	2.5501 × 10^−2^	irregular	Disrupted
DB9	3573	1 × 10^−3^	Integer multiples	Yes, Preserved
DB10	56,072	2.4 × 10^−3^	mostly irregular	Disrupted
DB11	40	5.684342 × 10^−14^	Mixed	Disrupted

^1^ Grid pattern: alignment of the eight difference levels following Δ_min_ with integer multiples of Δ_min_.

**Table 8:** Numerical indicators of observable quantization-grid preservation, including Δ_min_, P_0_, Egrid, and relative signal cardinality for each analyzed recording.

Dataset ID	Δ_min_	P_0_	Egrid	Card. Orig. %
DB1	3.0517578125 × 10^−4^	5.50125	1	0.1447%
DB2	9.43599809671153 × 10^−6^	0.0293	1	76.4746%
DB3	1.00 × 10^−5^	90.4038	1	0.2862%
DB4	1.004	5.83725	1	0.3110%
DB5	1.62 × 10^−7^	0.1375	0.005	15.3712%
DB6	1.512886517450740 × 10^−4^	0	0.003	100.0000%
DB7	8.05861 × 10^−4^	2.50788	1	3.1923%
DB8	0.025501000000000	2.4008	0.7595	7.3333%
DB9	0.001	4.39845	1	0.4444%
DB10	0.0024	55.5096	0.2151	0.6712%
DB11	5.6843460 × 10^−14^	0.00221	0.9924	99.2266%

**Table 9:** Signal-range and noise characteristics of the analyzed public sEMG datasets.

No	Max Value	Min Value	Robs	Baseline Noise RMS	RNR
1	0.3751 mV	−0.5685 mV	0.9436 mV	0.0026 mV	51.32 dB
2	0.2706 mV	−0.3478 mV	0.6184 mV	0.0369 mV	24.47 dB
3	0.0013	−0.0013	0.0026 V	1.459356783731186 × 10^−5^ V	45 dB
4	20	−26	46 mV	4.5503 mV	20.09 dB
5	0.0024	−0.0040	0.0063 vV	N/A ^1^	N/A
6	0.2651	−0.2238	0.4890 V	0.0320	23.67 dB
7	1.802710622710623	0.9686	0.8341 V	N/A ^1^	N/A
8	4.0250	−5.0536	9.0786 V	N/A ^1^	N/A
9	0.1650	−0.3200	0.4850 V	0.0396	21.76 dB
10	2.3999 V	0.0024 V	2.397 V	0.0032	57.50 dB
11	0.0012 V	−0.0014 V	0.0027 V	3.2311607 × 10^−6^ V	58.44 dB

^1^ Insufficient baseline data for reliable noise estimation.

**Table 10:** Observable effective resolution (OER) of the ABR and OAE signals for three participants in the reference dataset.

Participant	ABR OER (Bits)	OAE OER (Bits)
N1	15	22
N2	23	23
N3	21	23

ABR: Auditory Brainstem Response. OAE: Otoacoustic Emissions.

**Table 11:** Quantization-related indicators for the selected OAE recording from the reference dataset.

Signal	Δ_min_	P_0_ (%)	Egrid	Relative Cardinality (%)
OAE	1.19 × 10^−8^,	0.025119	1	18.5%

**Table 12:** Complementary interpretation of the numerical descriptors used to assess observable quantization-grid structure.

Descriptor	Numerical Property Characterized	Interpretation in This Study
$P_{0}$	Repetition of consecutive numerical values	Higher values may accompany a discrete numerical structure, but sample repetition alone does not establish grid preservation, as illustrated by DB10.
Relative signal cardinality	Numerical occupancy of the analyzed signal vector	Lower cardinality may accompany a discrete structure, but cardinality depends on signal dynamics and acquisition characteristics and is not by itself a criterion for grid preservation.
$E_{\mathrm{grid}}$	Approximate alignment of nonzero first differences with integer multiples of $\Delta_{\min}$	Quantifies alignment at the adopted tolerance (ε=0.001); a value close to or equal to unity is not by itself sufficient to establish an ordered grid pattern, as illustrated by DB2.
Difference-level pattern	Occurrence of observed nonzero $\left|\Delta x\right|$ levels at integer multiples of $\Delta_{\min}$	An ordered sequence of levels consistent with integer multiples of $\Delta_{\min}$ supports preservation of an observable discrete grid, whereas intermediate or irregularly spaced levels indicate disruption of that structure.

## Data Availability

This study did not generate new primary experimental data. All sEMG datasets analyzed (DB1–DB11) are publicly available third-party databases, accessed through the repositories cited in Table 1 and the References section (refs. [14,15,16,17,18,19,20,21,22,23,24,25,26,27,28,29,30]). The 24-bit reference dataset used for methodological validation (Section 2.5 and Section 3.4) is also publicly available on PhysioNet [36,37]. The derived quantization metrics (Δ_min_, OER, P0, cardinality, Egrid) computed by the authors for each dataset are reported in Table 6, Table 7, Table 8, Table 9, Table 10, Table 11 and Table 12, Appendix A (Table A1, Table A2, Table A3, Table A4, Table A5, Table A6, Table A7, Table A8, Table A9, Table A10, Table A11 and Table A12), and Appendix B (Table A13, Table A14, Table A15, Table A16, Table A17, Table A18, Table A19, Table A20, Table A21, Table A22 and Table A23). The relevant analysis scripts used to compute the numerical metrics reported in this study were developed locally and are provided as Supplementary Materials with this article.

## Supplementary Materials

The following supporting information can be downloaded at: https://www.mdpi.com/article/10.3390/s26216890/s1. Code S1: compute_indicators.m, MATLAB function for computing the numerical indicators described in the study; Code S2: run_database_indicators.m, MATLAB script for applying the analysis to the selected sEMG datasets; Code S3: resolution_validation.m, MATLAB script for the controlled 8-bit numerical validation; Code S4: Controlled_Quantization_Filtering.m, MATLAB script for evaluating the effects of post-acquisition filtering on quantization-grid preservation; File S1: README.md, instructions for data access, script execution, and expected results for Codes S1–S3; File S2: Controlled_Experiment_Report.pdf, methodology, numerical results, and figures of the controlled filtering experiment; File S3: README_Controlled_Filtering.txt, execution instructions and expected results for Code S4.

## Abbreviations

The following abbreviations are used in this manuscript:

sEMG	Surface electromyography
ADC	Analog-to-digital converter
DR	Declared resolution
OER	Observable effective resolution
ENOB	Effective number of bits
RNR	Range-to-noise ratio
RMS	Root mean square
ABR	Auditory brainstem response
OAE	Otoacoustic emission
WFDB	Waveform Database
DAQ	Data acquisition
USB	Universal Serial Bus
SNR	Signal-to-noise ratio

## Appendix A. First-Order Difference Levels of Selected Recordings

The tables in this appendix provide the complete numerical results supporting the quantization-grid analysis performed in this study, including the eleven public sEMG datasets and the controlled reference dataset used for validation. Each table begins with the frequency of zero first-order differences (ΔDV = 0), which is reported separately because it does not correspond to a quantization-grid level.

The remaining entries correspond to the first nine distinct nonzero first-order difference levels observed in the dataset. The smallest nonzero value defines the minimum difference, $\left|\min\Delta DV\right|$, which is interpreted as the minimum observable numerical spacing. Subsequent levels are examined to determine whether they follow successive integer multiples of $\left|\min\Delta DV\right|$, as expected for a preserved observable discrete structure.

For clarity, integer-multiple relationships are explicitly indicated whenever they are observed. The final Table A12 corresponds to the reference dataset used for validation and demonstrates that the proposed methodology correctly identifies the discrete quantization structure in an independently acquired 24-bit recording.

**Table A1:** Frequencies and resolutions per ΔDV level—DB1, Channel 4.

ΔDV	Frequency	Resolution (mV)
0	70,416	0
|minΔDV|	137,029	3.0517578125 × 10^−4^
2|minΔDV|	127,453	6.103515625 × 10^−4^
3|minΔDV|	113,394	9.1552734375 × 10^−4^
4|minΔDV|	99,227	1.220703125 × 10^−3^
5|minΔDV|	85,414	1.52587890625 × 10^−3^
6|minΔDV|	73,177	1.8310546875 × 10^−3^
7|minΔDV|	62,015	2.13623046875 × 10^−3^
8|minΔDV|	53,865	2.44140625 × 10^−3^
9|minΔDV|	45,780	2.74658203125 × 10^−3^

**Table A2:** Frequencies and resolutions per ΔDV level—DB2, Channel 1.

ΔDV	Frequency	Resolution (V)
0	3	0
|minΔDV|	1	9.43599809671153 × 10^−6^
—	1	9.435998096715 × 10^−6^
—	2	9.43599809671673 × 10^−6^
—	3	9.43599809671847 × 10^−6^
—	1	1.88719961934335 × 10^−5^
—	3	1.88719961934369 × 10^−5^
—	2	2.83079942901519 × 10^−5^
4|minΔDV|	1	3.77439923868461 × 10^−5^
—	1	3.77439923868669 × 10^−5^

**Table A3:** Frequencies and resolutions per ΔDV level—DB3, Channel 1.

ΔDV	Frequency	Resolution (V)
0	65,381	0
|minΔDV|	1578	1 × 10^−5^
2|minΔDV|	1335	2 × 10^−5^
3|minΔDV|	862	3 × 10^−5^
4|minΔDV|	543	4 × 10^−5^
5|minΔDV|	395	5 × 10^−5^
6|minΔDV|	265	6 × 10^−5^
7|minΔDV|	239	7 × 10^−5^
8|minΔDV|	165	8 × 10^−5^
9|minΔDV|	150	9 × 10^−5^

**Table A4:** Frequencies and resolutions per ΔDV level—DB4, Channel 1.

ΔDV	Frequency	Resolution (mV)
0	807	0
|minΔDV|	1612	1 × $10^{0}$
2|minΔDV|	1572	2 × $10^{0}$
3|minΔDV|	1500	3 × $10^{0}$
4|minΔDV|	1408	4 × $10^{0}$
5|minΔDV|	1221	5 × $10^{0}$
6|minΔDV|	1015	6 × $10^{0}$
7|minΔDV|	919	7 × $10^{0}$
8|minΔDV|	789	8 × $10^{0}$
9|minΔDV|	633	9 × $10^{0}$

**Table A5:** Frequencies and resolutions per ΔDV level—DB5, Channel 1.

ΔDV	Frequency	Resolution (mV)
0	110	0
|minΔDV|	51	1.62 × 10^−7^
—	152	1.621 × 10^−7^
2|minΔDV|	2	3.24 × 10^−7^
—	177	3.241 × 10^−7^
—	44	3.242 × 10^−7^
—	7	4.861 × 10^−7^
—	215	4.862 × 10^−7^
—	2	4.863 × 10^−7^
—	52	6.482 × 10^−7^

**Table A6:** Frequencies and resolutions per ΔDV level—DB6, Channel 1.

ΔDV	Frequency	Resolution (mV)
0	0	0
|minΔDV|	1	1.51288651745074 × 10^−4^
—	1	2.56128808135372 × 10^−4^
—	1	2.99521765533444 × 10^−4^
—	1	3.5078515799237 × 10^−4^
—	1	3.86390034158291 × 10^−4^
—	1	4.03230260137364 × 10^−4^
—	1	4.67520362938442 × 10^−4^
—	1	4.99085281175934 × 10^−4^
—	1	5.97942613379018 × 10^−4^

**Table A7:** Frequencies and resolutions per ΔDV level—DB7, Channel 1.

ΔDV	Frequency	Resolution (V)
0	326	0
|minΔDV|	522	8.05861 × 10^−4^
2|minΔDV|	512	1.611722 × 10^−3^
3|minΔDV|	481	2.417582 × 10^−3^
4|minΔDV|	472	3.223443 × 10^−3^
5|minΔDV|	482	4.029304 × 10^−3^
6|minΔDV|	425	4.835165 × 10^−3^
7|minΔDV|	372	5.641026 × 10^−3^
8|minΔDV|	334	6.446886 × 10^−3^
9|minΔDV|	365	7.252747 × 10^−3^

**Table A8:** Frequencies and resolutions per ΔDV level—DB8, Channel 1.

ΔDV	Frequency	Resolution (mV)
0	72	0
|minΔDV|	39	2.5501 × 10^−2^
—	70	2.5502 × 10^−2^
—	79	5.1003 × 10^−2^
—	34	5.1004 × 10^−2^
—	13	7.6504 × 10^−2^
—	87	7.6505 × 10^−2^
—	49	1.02006 × 10^−1^
—	48	1.02007 × 10^−1^
—	89	1.27508 × 10^−1^

**Table A9:** Frequencies and resolutions per ΔDV level—DB9, Channel 1.

ΔDV	Frequency	Resolution (mV)
0	3573	0
|minΔDV|	6861	1 × 10^−3^
2|minΔDV|	6527	2 × 10^−3^
3|minΔDV|	6141	3 × 10^−3^
4|minΔDV|	5396	4 × 10^−3^
5|minΔDV|	4888	5 × 10^−3^
6|minΔDV|	4312	6 × 10^−3^
7|minΔDV|	3834	7 × 10^−3^
8|minΔDV|	3344	8 × 10^−3^
9|minΔDV|	2986	9 × 10^−3^

**Table A10:** Frequencies and resolutions per ΔDV level—DB10, Channel 1.

ΔDV	Frequency	Resolution (mV)
0	56,072	0
|minΔDV|	8302	2.4 × 10^−3^
—	11,826	2.5 × 10^−3^
2|minΔDV|	1357	4.8 × 10^−3^
—	8761	4.9 × 10^−3^
—	3672	7.3 × 10^−3^
—	1369	7.4 × 10^−3^
—	912	9.7 × 10^−3^
—	1875	9.8 × 10^−3^
—	1522	1.22 × 10^−2^

**Table A11:** Frequencies and resolutions per ΔDV level—DB11, Channel 1.

ΔDV	Frequency	Resolution (V)
0	40	0
|minΔDV|	2	5.684342 × 10^−14^
—	1	7.105427 × 10^−14^
2|minΔDV| ^1^	5	* 1.136868 × 10^−13^
—	1	1.421085 × 10^−13^
—	1	1.563194 × 10^−13^
—	1	1.634248 × 10^−13^
3|minΔDV| ^1^	2	* 1.705303 × 10^−13^
4|minΔDV| ^1^	20	* 2.273737 × 10^−13^
—	3	2.842171 × 10^−13^

^1^: * Values are not exact integer multiples of Δ_min_ but agree with the corresponding multiples within a tolerance of ±4 × 10^−20^.

**Table A12:** Frequencies and corresponding resolutions of the first-order difference levels for the 24-bit reference dataset (Evoked Auditory Responses in Normals, OAE channel), used for methodological validation.

ΔDV	Frequency	Resolution (V)
0	521	0
|minΔDV|	952	1.19 × 10^−8^
2|minΔDV|	943	2.38 × 10^−8^
3|minΔDV|	961	3.58 × 10^−8^
4|minΔDV|	954	4.77 × 10^−8^
5|minΔDV|	956	5.96 × 10^−8^
6|minΔDV|	900	7.15 × 10^−8^
7|minΔDV|	995	8.34 × 10^−8^
8|minΔDV|	919	9.54 × 10^−8^
9|minΔDV|	1018	1.073 × 10^−7^

ΔDV = |n × minΔDV|, n = 1, 2, 3…; Samples: 2,074,112 | Fs: 48,000 Hz | ADC: Lynx Two 24-bit.

## Appendix B. Within-Dataset Consistency Assessment Across Participants and Channels

Channel identifiers follow the column indices of the distributed data matrices. For datasets in which the first column contains time information, the first sEMG channel corresponds to column 2.

**Table A13:** Quantization indicators of DB1 (BIOPAC MP36) per participant and channel.

Part.	Ch.	Δ_min_	*P*_0_ (%)	*E*_grid_	Card. (%)	OER (Bits)	*N*
1	2	3.052 × 10^−4^	1.358	1	0.56	13.48	1,280,000
1	3	3.052 × 10^−4^	1.931	1	0.54	13.04	1,280,000
1	4	3.052 × 10^−4^	5.501	1	0.14	11.59	1,280,000
2	2	3.052 × 10^−4^	1.392	1	1.44	14.31	1,280,000
2	3	3.052 × 10^−4^	1.946	1	0.77	13.34	1,280,000
2	4	3.052 × 10^−4^	6.773	1	0.20	12.26	1,280,000
3	2	3.052 × 10^−4^	19.041	1	1.63	14.57	1,280,000
3	3	3.052 × 10^−4^	2.696	1	0.70	13.99	1,280,000
3	4	3.052 × 10^−4^	6.586	1	0.15	12.62	1,280,000
4	2	3.052 × 10^−4^	1.426	1	0.53	12.97	1,280,000
4	3	3.052 × 10^−4^	1.280	1	0.39	13.12	1,280,000
4	4	3.052 × 10^−4^	5.988	1	0.17	12.02	1,280,000
5	2	3.052 × 10^−4^	1.200	1	0.72	13.32	1,280,000
5	3	3.052 × 10^−4^	1.595	1	0.37	12.92	1,280,000
5	4	3.052 × 10^−4^	5.572	1	0.18	12.13	1,280,000

Part. = participant; Ch. = channel (column of the signal file); Card. = relative signal cardinality; OER in bits; *N* = number of samples. Values are rounded for presentation; calculations used full precision.

**Table A14:** Quantization indicators of DB2 (GRABMyo) per participant and channel.

Part.	Ch.	Δ_min_	*P*_0_ (%)	*E*_grid_	Card. (%)	OER (Bits)	*N*
1	1	9.436 × 10^−6^	0.029	1	76.47	16.00	10,240
1	2	8.813 × 10^−6^	0.020	1	77.76	16.00	10,240
1	3	9.774 × 10^−6^	0.049	1	78.14	16.00	10,240
2	1	2.084 × 10^−6^	0	1	82.33	16.00	10,240
2	2	2.061 × 10^−6^	0.0098	1	83.45	16.00	10,240
2	3	2.499 × 10^−6^	0.0098	1	82.76	16.00	10,240
3	1	4.284 × 10^−6^	0.0098	1	83.37	16.00	10,240
3	2	4.359 × 10^−6^	0.0098	1	84.21	16.00	10,240
3	3	4.930 × 10^−6^	0.020	1	83.99	16.00	10,240
4	1	6.468 × 10^−6^	0	1	82.06	16.00	10,240
4	2	6.782 × 10^−6^	0.039	1	81.63	16.00	10,240
4	3	7.147 × 10^−6^	0.029	1	81.04	16.00	10,240
5	1	5.158 × 10^−6^	0	1	83.10	16.00	10,240
5	2	4.999 × 10^−6^	0.0098	1	83.82	16.00	10,240
5	3	5.680 × 10^−6^	0.020	1	83.41	16.00	10,240

Part. = participant; Ch. = channel (column of the signal file); Card. = relative signal cardinality; OER in bits; *N* = number of samples. Values are rounded for presentation; calculations used full precision.

**Table A15:** Quantization indicators of DB3 (MYO Thalmic armband) per participant and channel.

Part.	Ch.	Δ_min_	*P*_0_ (%)	*E*_grid_	Card. (%)	OER (Bits)	*N*
1	2	1 × 10^−5^	90.404	1	0.29	7.99	72,322
1	3	1 × 10^−5^	90.133	1	0.12	7.32	72,322
1	4	1 × 10^−5^	89.999	1	0.18	7.53	72,322
2	2	1 × 10^−5^	90.548	1	0.25	7.89	63,196
2	3	1 × 10^−5^	90.127	1	0.23	7.84	63,196
2	4	1 × 10^−5^	90.025	1	0.21	7.77	63,196
3	2	1 × 10^−5^	89.634	1	0.38	7.99	56,568
3	3	1 × 10^−5^	89.197	1	0.42	7.99	56,568
3	4	1 × 10^−5^	88.939	1	0.26	7.80	56,568
4	2	1 × 10^−5^	90.923	1	0.36	7.97	59,107
4	3	1 × 10^−5^	90.740	1	0.20	7.43	59,107
4	4	1 × 10^−5^	90.608	1	0.23	7.43	59,107
5	2	1 × 10^−5^	90.775	1	0.25	7.92	57,118
5	3	1 × 10^−5^	90.313	1	0.19	7.61	57,118
5	4	1 × 10^−5^	90.129	1	0.14	7.18	57,118

Part. = participant; Ch. = channel (column of the signal file); Card. = relative signal cardinality; OER in bits; *N* = number of samples. Values are rounded for presentation; calculations used full precision.

**Table A16:** Quantization indicators of DB4 (MYO Thalmic armband) per channel (single subject).

Part.	Ch.	Δ_min_	*P*_0_ (%)	*E*_grid_	Card. (%)	OER (Bits)	*N*
1	2	1	5.837	1	0.31	5.52	13,826
1	3	1	15.783	1	0.12	4.00	13,826
1	4	1	10.647	1	0.20	4.75	13,826
1	5	1	15.870	1	0.16	4.46	13,826
1	6	1	14.604	1	0.12	4.00	13,826
1	7	1	14.604	1	0.12	4.00	13,826
1	8	1	14.033	1	0.14	4.25	13,826
1	9	1	5.208	1	0.43	6.04	13,826

Part. = participant; Ch. = channel (column of the signal file); Card. = relative signal cardinality; OER in bits; *N* = number of samples. Values are rounded for presentation; calculations used full precision.

**Table A17:** Quantization indicators of DB5 (Delsys Bagnoli-8) per participant and channel.

Part.	Ch.	Δ_min_	*P*_0_ (%)	*E*_grid_	Card. (%)	OER (Bits)	*N*
1	1	1.620 × 10^−7^	0.138	0.0050	15.37	15.25	80,000
1	2	1.620 × 10^−7^	0.218	0.0074	10.30	14.12	80,000
1	3	1.620 × 10^−7^	0.398	0.0159	4.33	12.76	80,000
2	1	1.620 × 10^−7^	0.255	0.0105	5.57	13.18	80,000
2	2	1.620 × 10^−7^	0.324	0.0132	6.66	13.42	80,000
2	3	1.620 × 10^−7^	0.754	0.0300	1.74	11.14	80,000
3	1	1.620 × 10^−7^	0.075	0.0035	13.61	14.44	80,000
3	2	1.620 × 10^−7^	0.121	0.0053	11.27	14.07	80,000
3	3	1.620 × 10^−7^	0.238	0.0091	6.52	13.06	80,000
4	1	1.620 × 10^−7^	0.104	0.0041	16.50	15.06	80,000
4	2	1.620 × 10^−7^	0.109	0.0050	17.94	15.24	80,000
4	3	1.620 × 10^−7^	0.363	0.0153	3.97	12.46	80,000
5	1	1.620 × 10^−7^	0.136	0.0047	10.09	13.28	80,000
5	2	1.620 × 10^−7^	0.269	0.0102	6.51	12.57	80,000
5	3	1.620 × 10^−7^	0.506	0.0193	3.23	11.66	80,000

Part. = participant; Ch. = channel (column of the signal file); Card. = relative signal cardinality; OER in bits; *N* = number of samples. Values are rounded for presentation; calculations used full precision.

**Table A18:** Quantization indicators of DB6 (CapgMyo) per participant and channel.

Part.	Ch.	Δ_min_	*P*_0_ (%)	*E*_grid_	Card. (%)	OER (Bits)	*N*
1	1	1.513 × 10^−4^	0	0.0030	100	11.66	1000
1	2	5.433 × 10^−6^	0	0.0010	100	16.60	1000
1	3	4.428 × 10^−5^	0	0.0020	100	13.61	1000
2	1	4.814 × 10^−6^	0	0.0030	100	15.39	1000
2	2	2.270 × 10^−6^	0	0.0040	100	16.74	1000
2	3	4.572 × 10^−5^	0	0.0050	100	12.42	1000
3	1	4.557 × 10^−7^	0	0.0010	100	16.43	1000
3	2	2.593 × 10^−6^	0	0.0030	100	13.83	1000
3	3	1.200 × 10^−5^	0	0.0010	100	12.79	1000
4	1	9.094 × 10^−6^	0	0.0030	100	15.22	1000
4	2	6.175 × 10^−6^	0	0.0040	100	14.59	1000
4	3	5.289 × 10^−5^	0	0.0030	100	12.11	1000
5	1	4.922 × 10^−6^	0	0.0040	100	14.34	1000
5	2	2.322 × 10^−6^	0	0.0020	100	16.21	1000
5	3	8.162 × 10^−6^	0	0.0030	100	14.14	1000

Part. = participant; Ch. = channel (column of the signal file); Card. = relative signal cardinality; OER in bits; *N* = number of samples. Values are rounded for presentation; calculations used full precision.

**Table A19:** Quantization indicators of DB7 (WyoFlex armband) per participant and channel.

Part.	Ch.	Δ_min_	*P*_0_ (%)	*E*_grid_	Card. (%)	OER (Bits)	*N*
1	1	8.059 × 10^−4^	2.508	1	3.19	10.02	13,000
1	2	8.059 × 10^−4^	3.854	1	1.43	8.23	13,000
1	3	8.059 × 10^−4^	13.055	1	2.13	9.26	13,000
2	1	8.059 × 10^−4^	3.416	1	1.88	8.48	13,000
2	2	8.059 × 10^−4^	3.693	1	2.45	9.31	13,000
2	3	8.059 × 10^−4^	12.563	1	4.16	10.49	13,000
3	1	8.059 × 10^−4^	4.954	1	1.77	8.96	13,000
3	2	8.059 × 10^−4^	14.632	1	0.98	7.78	13,000
3	3	8.059 × 10^−4^	17.071	1	1.57	8.80	13,000
4	1	8.059 × 10^−4^	4.231	1	1.98	9.54	13,000
4	2	8.059 × 10^−4^	11.832	1	1.08	7.96	13,000
4	3	8.059 × 10^−4^	14.955	1	1.79	9.00	13,000
5	1	8.059 × 10^−4^	2.800	1	2.12	8.83	13,000
5	2	8.059 × 10^−4^	3.839	1	1.45	8.27	13,000
5	3	8.059 × 10^−4^	9.139	1	1.98	8.38	13,000

Part. = participant; Ch. = channel (column of the signal file); Card. = relative signal cardinality; OER in bits; *N* = number of samples. Values are rounded for presentation; calculations used full precision.

**Table A20:** Quantization indicators of DB8 (NI USB-009) per participant and channel.

Part.	Ch.	Δ_min_	*P*_0_ (%)	*E*_grid_	Card. (%)	OER (Bits)	*N*
1	1	2.550 × 10^−2^	2.401	0.7595	7.33	8.48	3000
1	2	2.551 × 10^−2^	3.568	0.9627	2.73	6.67	3000
2	1	2.550 × 10^−2^	2.968	0.8409	6.40	8.21	3000
2	2	2.551 × 10^−2^	2.001	0.7179	7.43	8.16	3000
3	1	2.550 × 10^−2^	6.869	0.9986	2.03	6.39	3000
3	2	2.551 × 10^−2^	5.168	0.9631	3.87	7.35	3000
4	1	2.550 × 10^−2^	4.635	0.9997	1.97	6.13	3000
4	2	2.551 × 10^−2^	4.935	0.9916	2.77	6.82	3000
5	1	2.550 × 10^−2^	7.503	0.9996	1.70	5.83	3000
5	2	2.551 × 10^−2^	3.068	0.8875	4.03	7.27	3000

Part. = participant; Ch. = channel (column of the signal file); Card. = relative signal cardinality; OER in bits; *N* = number of samples. Values are rounded for presentation; calculations used full precision.

**Table A21:** Quantization indicators of DB9 (NI USB-6212) per participant and channel.

Part.	Ch.	Δ_min_	*P*_0_ (%)	*E*_grid_	Card. (%)	OER (Bits)	*N*
1	1	1 × 10^−3^	4.398	1	0.44	8.92	81,234
1	2	1 × 10^−3^	2.881	1	1.08	10.41	81,234
1	3	1 × 10^−3^	2.693	1	0.69	9.72	81,234
2	1	1 × 10^−3^	4.848	1	0.23	8.10	83,348
2	2	1 × 10^−3^	5.151	1	0.26	8.54	83,348
2	3	1 × 10^−3^	5.508	1	0.21	7.80	83,348
3	1	3.280 × 10^−4^	1.571	0.0044	0.92	10.79	89,703
3	2	3.280 × 10^−4^	1.312	0.0042	1.18	10.63	89,703
3	3	3.280 × 10^−4^	1.816	0.0052	0.83	11.47	89,703
4	1	3.280 × 10^−4^	2.007	0.0064	0.86	10.42	82,475
4	2	3.280 × 10^−4^	2.803	0.0079	0.60	10.92	82,475
4	3	3.280 × 10^−4^	3.253	0.0100	0.63	9.88	82,475
5	1	3.280 × 10^−4^	1.197	0.0033	1.11	12.20	98,518
5	2	3.280 × 10^−4^	1.345	0.0040	1.19	11.60	98,518
5	3	3.280 × 10^−4^	1.377	0.0046	0.77	11.12	98,517

Part. = participant; Ch. = channel (column of the signal file); Card. = relative signal cardinality; OER in bits; *N* = number of samples. Values are rounded for presentation; calculations used full precision.

**Table A22:** Quantization indicators of DB10 (NinaPro DB1, Otto Bock) per participant and channel.

Part.	Ch.	Δ_min_	*P*_0_ (%)	*E*_grid_	Card. (%)	OER (Bits)	*N*
1	1	2.400 × 10^−3^	55.510	0.2151	0.67	9.96	101,014
1	2	2.400 × 10^−3^	76.457	0.0600	0.81	10.01	101,014
1	3	2.400 × 10^−3^	67.818	0.0833	0.39	9.12	101,014
2	1	2.400 × 10^−3^	11.776	0.0847	1.15	10.65	100,686
2	2	2.400 × 10^−3^	14.313	0.0488	0.69	9.81	100,686
2	3	2.400 × 10^−3^	23.822	0.0986	0.50	9.14	100,686
3	1	2.400 × 10^−3^	58.125	0.2022	0.57	9.41	100,720
3	2	2.400 × 10^−3^	67.132	0.0673	0.56	9.44	100,720
3	3	2.400 × 10^−3^	56.192	0.0753	0.50	9.18	100,720
4	1	2.400 × 10^−3^	26.112	0.1710	0.74	9.90	100,835
4	2	2.400 × 10^−3^	69.516	0.1383	0.26	8.25	100,835
4	3	2.400 × 10^−3^	63.004	0.1179	0.41	9.13	100,835
5	1	2.400 × 10^−3^	89.687	0.1728	0.0079	2.83	100,894
5	2	2.400 × 10^−3^	17.098	0.0831	0.60	9.65	100,894
5	3	2.400 × 10^−3^	26.786	0.1204	1.71	10.92	100,894

Part. = participant; Ch. = channel (column of the signal file); Card. = relative signal cardinality; OER in bits; *N* = number of samples. Values are rounded for presentation; calculations used full precision.

**Table A23:** Quantization indicators of DB11 (NinaPro DB2, Delsys Trigno) per participant and channel.

Part.	Ch.	Δ_min_	*P*_0_ (%)	*E*_grid_	Card. (%)	OER (Bits)	*N*
1	1	5.684 × 10^−14^	0.0022	0.9924	99.23	35.45	1,808,331
1	2	1.421 × 10^−14^	0.0022	0.9995	99.23	37.04	1,808,331
1	3	1.205 × 10^−21^	2.725	>0.9999	23.16	62.99	1,808,331
2	1	2.274 × 10^−13^	0.0011	0.9709	99.19	33.36	1,803,711
2	2	1.776 × 10^−13^	0.0084	0.8712	98.55	34.18	1,803,711
2	3	4.016 × 10^−22^	2.352	>0.9999	16.54	62.55	1,803,711
3	1	3.638 × 10^−12^	0.00028	0.5298	99.12	31.46	1,795,150
3	2	1.137 × 10^−13^	0.00056	0.9873	99.03	34.80	1,795,150
3	3	8.031 × 10^−23^	2.181	>0.9999	27.65	65.05	1,795,150
4	1	1.137 × 10^−13^	0.0014	0.9927	99.17	34.54	1,797,647
4	2	1.279 × 10^−13^	0.00056	0.8575	99.17	33.36	1,797,647
4	3	2.008 × 10^−23^	3.185	>0.9999	6.71	65.33	1,797,647
5	1	2.501 × 10^−12^	0	0.2006	99.20	32.27	1,801,513
5	2	8.882 × 10^−15^	0.0027	0.9625	99.10	38.36	1,801,513
5	3	4.016 × 10^−23^	4.360	>0.9999	8.74	65.74	1,801,513

Part. = participant; Ch. = channel (column of the signal file); Card. = relative signal cardinality; OER in bits; *N* = number of samples. Egrid values reported as >0.9999 are below 1 but round to 1.0000 at four decimals. Values are rounded for presentation; calculations used full precision.

## References

[1] Texas Instruments (2017). ADS1299-x Low-Noise, 4-, 6-, 8-Channel, 24-Bit Analog-to-Digital Converter for EEG and Biopotential Measurements.

[2] Analog Devices, Inc EEG Measurement Solutions; Application and Signal-Chain Documentation (Web Page). https://www.analog.com/en/solutions/healthcare/vital-signs-measurements/eeg-measurement.html.

[3] Xu J., Qu T., Pan Q., Li Y., Liu L., Li Y., Zhou J., Yao C., Hong Z. (2025). Analog Front-End Circuit Techniques for Wearable ExG, BioZ, and PPG Signal Acquisition: A Review. IEEE Open J. Solid-State Circuits Soc..

[4] Inafuco A.T.P., Machoski P., Campos D.P., Pichorim S.F., Mendes Junior J.J.A. (2025). MOT: A Low-Latency, Multichannel Wireless Surface Electromyography Acquisition System Based on the AD8232 Front-End. Sensors.

[5] Semmlow J. (2024). Circuits, Signals, and Systems for Bioengineers: A MATLAB-Based Introduction.

[6] Verdini F., Tigrini A., Scattolini M., Mobarak R., Fioretti S., Burattini L., Mengarelli A. (2026). Impact of Sampling Frequency and Signal Quantization on Myoelectric-Based Hand Gesture Recognition. Biomed. Signal Process. Control.

[7] Ortiz-Catalan M. (2015). Cardinality as a highly descriptive feature in myoelectric pattern recognition for decoding motor volition. Front. Neurosci..

[8] Shinde A., Shete V., Mehendale N. (2026). Optimized Signal Acquisition and Advanced AI for Robust 1D EMG Classification: A Comparative Study of Machine Learning, Deep Learning, and Reinforcement Learning. Bioengineering.

[9] Halford J.J., Sabau D., Drislane F.W., Tsuchida T.N., Sinha S.R. (2016). American Clinical Neurophysiology Society Guideline 4: Recording Clinical EEG on Digital Media. Neurodiagnostic J..

[10] Niso G., Romero E., Moreau J.T., Araujo A., Krol L.R. (2023). Wireless EEG: A Survey of Systems and Studies. NeuroImage.

[11] Al-Ayyad M., Owida H.A., De Fazio R., Al-Naami B., Visconti P. (2023). Electromyography Monitoring Systems in Rehabilitation: A Review of Clinical Applications, Wearable Devices and Signal Acquisition Methodologies. Electronics.

[12] Frey S., Spacone G., Cossettini A., Guermandi M., Schilk P., Benini L., Kartsch V. (2026). BioGAP-Ultra: A Modular Edge-AI Platform for Wearable Multimodal Biosignal Acquisition and Processing. IEEE Trans. Biomed. Circuits Syst..

[13] Bravo-Zanoguera M.E., Gomez-Caraveo A., Amezquita-Garcia J.A., Murrieta-Rico F.N., Yañez G.G., González D.C. (2026). Assessing High-Resolution Integrity in Open sEMG Datasets for Machine Learning Applications. Proceedings of the 2026 Global Medical Engineering Physics Exchanges/Pan American Health Care Exchanges (GMEPE/PAHCE), Rio de Janeiro, Brazil, 23–28 March 2026.

[14] Ozdemir M.A., Kisa D.H., Guren O., Akan A. (2021). Dataset for Multi-Channel Surface Electromyography (sEMG) Signals of Hand Gestures. Mendeley Data.

[15] Ozdemir M.A., Kisa D.H., Guren O., Akan A. (2022). Dataset for multi-channel surface electromyography (sEMG) signals of hand gestures. Data Brief.

[16] Jiang N., Pradhan A., He J. (2024). Gesture Recognition and Biometrics ElectroMyogram (GRABMyo). PhysioNet.

[17] Pradhan A., He J., Jiang N. (2022). Multi-day dataset of forearm and wrist electromyogram for hand gesture recognition and biometrics. Sci. Data.

[18] Lobov S., Krilova N., Kastalskiy I., Kazantsev V., Makarov V.A. (2018). EMG Data for Gestures [Dataset]. UCI Mach. Learn. Repos..

[19] Khan A.M., Khawaja S.G., Akram M.U., Khan A.S. (2020). SEMG dataset of routine activities. Mendeley Data.

[20] Khan A.M., Khawaja S.G., Akram M.U., Khan A.S. (2020). SEMG Dataset of Routine Activities. Data Brief.

[21] Khushaba R.N. Biosignals Repository. https://rami-khushaba.com/biosignals-repository.

[22] Geng W., Du Y., Jin W., Wei W., Hu Y., Li J. (2016). Data from: Gesture Recognition by Instantaneous Surface EMG Images (CapgMyo-DBa). figshare.

[23] Geng W., Du Y., Jin W., Wei W., Hu Y., Li J. (2016). Gesture recognition by instantaneous surface EMG images. Sci. Rep..

[24] Gomez-Correa M., Ballesteros M., Salgado I., Cruz-Ortiz D. (2023). Forearm sEMG data from young healthy humans during the execution of hand movements. Figshare. Collect..

[25] Gomez-Correa M., Ballesteros M., Salgado I., Cruz-Ortiz D. (2023). Forearm sEMG data from young healthy humans during the execution of hand movements. Sci. Data.

[26] Sapsanis C., Georgoulas G., Tzes A., Lymberopoulos D. (2013). SEMG for Basic Hand Movements Data Set. UCI Mach. Learn. Repos..

[27] Miljković N., Isaković M.S. (2020). Surface electromyogram (sEMG) dataset recorded from forearm for 9 hand movements and three electrode array positions. Zenodo.

[28] Ninapro DB1 Guidelines. https://ninapro.hevs.ch/instructions/DB1.html.

[29] Atzori M., Gijsberts A., Castellini C., Caputo B., Hagenberg A.G., Elsig S., Giatsidis G., Bassetto F., Müller H. (2014). Electromyography data for non-invasive naturally-controlled robotic hand prostheses. Sci. Data.

[30] Ninapro DB2 Guidelines. https://ninapro.hevs.ch/instructions/DB2.html.

[31] OT Bioelettronica (2013). EMG-USB2+ User Manual V1.3.

[32] Sapsanis C. (2013). Recognition of Basic Hand Movements Using Electromyography. Diploma Thesis.

[33] National Instruments (2026). USB-6009 Specifications.

[34] Pizzolato S., Tagliapietra L., Cognolato M., Reggiani M., Müller H., Atzori M. (2017). Comparison of six electromyography acquisition setups on hand movement classification tasks. PLoS ONE.

[35] Delsys Inc (2024). Trigno® Wireless Biofeedback System User’s Guide (MAN-049-1-1).

[36] Silva I., Epstein M. (2011). Evoked Auditory Responses in Normals.

[37] Silva I., Epstein M. (2010). Estimating loudness growth from tone-burst evoked responses. J. Acoust. Soc. Am..

[38] White P. (2002). Lynx Studio Technology Lynx Two: PCI Multichannel Audio Interface.

[39] Oppenheim A.V., Weinstein C.J. (1972). Effects of Finite Register Length in Digital Filtering and the Fast Fourier Transform. Proc. IEEE.

