# Supplementary material for: Assessing Observable Resolution in Open-Access sEMG Datasets for High-Resolution Hand-Gesture Research: A Quantization-Aware Methodological Study
Source: Sensors (Basel). 2026 Oct 30;26(21):6890. doi: 10.3390/s26216890 (PMC13766169; doi:10.3390/s26216890)
Supplement: Supplementary file 1 [file sensors-26-06890-s001.zip › Controlled_Experiment_Report.pdf]

Controlled Experiment: Post-Acquisition Filtering and the Quantization Grid

This experiment checks whether filtering after acquisition breaks the grid, and how the indicators used in the article (especially E_{grid}) respond to it, compared with the deviation from the known original grid. A synthetic 8-bit signal is compared under three conditions: unprocessed, band-pass filtered (non-integer coefficients) and filtered with integer coefficients.

1 Method

1. **Synthetic analog signal.** $s[n] = 0.65\sin(2\pi\,35n/f_s) + 0.15\sin(2\pi\,90n/f_s) + 0.12\,\varepsilon[n]$, with Gaussian noise $\varepsilon[n]$, $N = 10\,000$ samples and $f_s = 1$ kHz (10 s). The seed `rng(1,'twister')` makes the noise reproducible. The signal is clipped to $[-1, 1]$.
2. **Signed 8-bit quantizer.** $q = 2/2^8 = 1/128$, integer codes $k = \text{round}(s/q)$ clipped to $[-128, 127]$, and $x = k\,q$. By construction, x lies exactly on the ADC grid.
3. **Three conditions.**
   - **Original 8-bit**: x without processing (reference).
   - **Band-pass FIR**: a 201-tap windowed-sinc filter (Hamming window) for 20–150 Hz, applied with `conv(x,h,'same')`. Its coefficients are non-integer.
   - **Integer FIR control**: $y[n] = (k[n] - k[n-1])\,q$, computed on the integer codes. Integer coefficients cannot move a sample off the grid. This operation serves as a grid-preserving arithmetic control rather than a frequency-response-matched counterpart to the band-pass filter.
4. **Metrics.**

Metric	Definition
$\Delta_{\min}$	Smallest nonzero $\lvert x[n] - x[n-1]\rvert$.
P_0 (%)	Share of consecutive differences equal to zero.
Card_{abs} / Card (%)	Number of distinct values, absolute and as a percentage of N. The 8-bit maximum is 256.
E_{grid}	Proportion of nonzero first differences approximately aligned with integer multiples of $\Delta_{\min}$, using a tolerance of 10^{-3}. It is computed *blindly*, without knowledge of q.
Max. deviation from original grid (LSB)	$\max_n \lvert x[n]/q - \text{round}(x[n]/q)\rvert$: the distance to the *original* grid, where 0 means on the grid and 0.5 is the worst case. It requires the true q.
RD, OER (bits)	$\text{RD} = \max x - \min x$; $\text{OER} = \log_2(\text{RD}/\Delta_{\min})$.

2 Script

The experiment is implemented in `Controlled_Quantization_Filtering.m`. It was tested in MATLAB R2026a and requires no toolboxes. The script generates the signal, applies the filters, prints the results table in the Command Window (also saved as `Filtering_Quantization_Results.csv`) and exports the two figures shown in Section 3. To reproduce the results, place the script in any folder and run it; the full `.m` file is provided as supplementary material.

3 Results

Table 1: Results obtained in MATLAB ($N = 10\,000$, $q = 2/2^8 = 0.0078125$).

Condition	Δ_{min}	P_0 (%)	Card_{abs}	Card (%)	E_{grid}	Max. dev. from original grid (LSB)	RD	OER (bits)
**Original 8-bit**	0.0078125	1.5502	255	2.55	**1**	**0**	1.9922	7.9944
**Band-pass FIR**	8.5159×10^{-6}	0	**10000**	**100**	**0.0018002**	**0.49999**	1.8541	**17.732**
**Integer FIR control**	0.0078125	0.92009	171	1.71	**1**	**0**	1.5469	7.6294

The Card_{abs} of 10 000 for the band-pass FIR refers to the numerical representation of the signal *after* filtering (one distinct value per sample), not to the output of an ADC with higher physical resolution; the acquisition itself remains 8-bit, with at most 256 codes. Likewise, Card (%) is relative to the 10 000 analysed samples, not to the number of values representable in floating point.

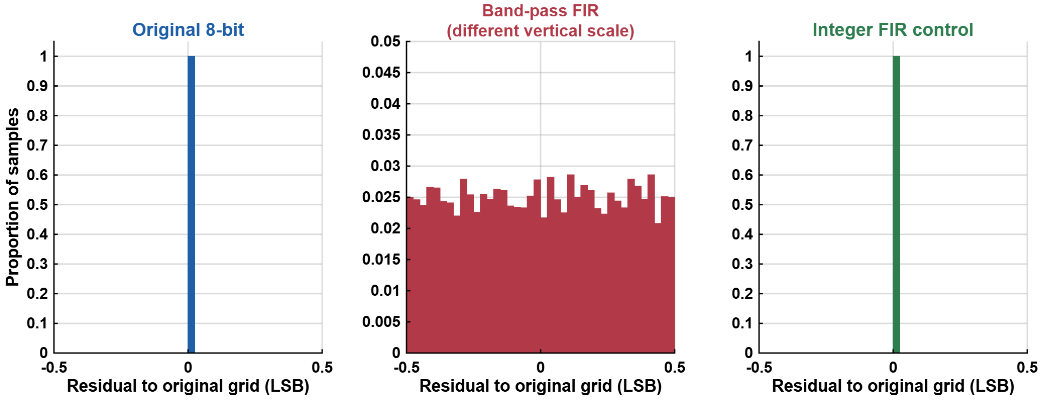

Figure 1: Distribution of the residual of each sample with respect to the original 8-bit grid. The original signal and the integer control lie entirely at zero. The band-pass FIR spreads the residuals over $[-0.5, 0.5]$ LSB. Note that the central panel uses a different vertical scale so that this distribution can be seen.

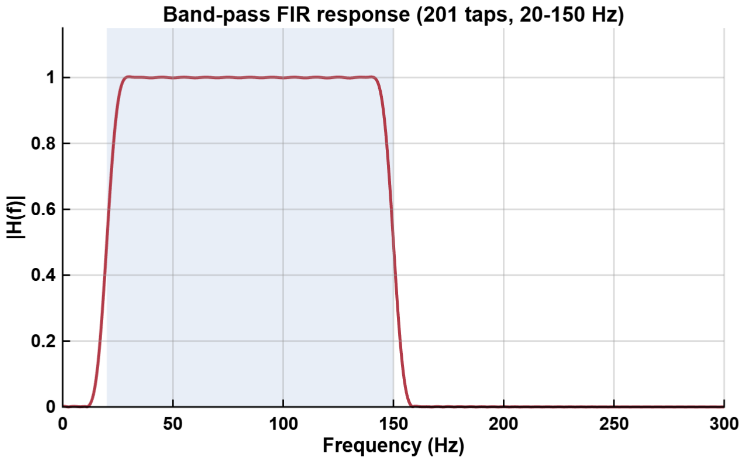

Figure 2: Magnitude response of the 201-tap band-pass FIR. The shaded region marks the 20–150 Hz pass band.

4 Discussion and conclusion

The original 8-bit signal behaves as expected: $\Delta_{\text{min}} = q$, $E_{\text{grid}} = 1$, zero deviation from the original grid and an OER of about 8 bits. The integer-coefficient filter gives the same picture, because subtracting two grid values always yields another grid value; this holds because the operation is

applied directly to the integer codes, without fractional coefficients or subsequent normalization, so the quantization structure is preserved.

The band-pass filter behaves differently. Its 201 coefficients combine neighbouring samples, and in this implementation every output sample becomes a distinct numerical value. The residuals with respect to the original grid spread over ± 0.5 LSB and E_{grid} drops to 0.0018. Although OER increased to approximately 17.7 bits, the known 8-bit acquisition resolution remained unchanged. The increase reflects finer numerical spacing introduced by the filtering operation rather than additional ADC resolution.

These results show that digital filtering does not necessarily disrupt the quantization grid. The numerical effects depend on the filter coefficients and their arithmetic implementation. In this controlled experiment, the grid-alignment indicator was consistent with the independently calculated deviations from the known original quantization grid. This agreement supports its use as a complementary descriptor of observable numerical structure, without implying that it can identify all forms of post-acquisition processing.
